# The Scottish COVID Cancer Immunity Prevalence Study: A Longitudinal Study of SARS-CoV-2 Immune Response in Patients Receiving Anti–Cancer Treatment

**DOI:** 10.1093/oncolo/oyac257

**Published:** 2023-01-31

**Authors:** Karin Purshouse, John P Thomson, Mahéva Vallet, Lorna Alexander, Isaac Bonisteel, Maree Brennan, David A Cameron, Jonine D Figueroa, Elizabeth Furrie, Pamela Haig, Mattea Heck, Hugh McCaughan, Paul Mitchell, Heather McVicars, Lorraine Primrose, Ines Silva, Kate Templeton, Natalie Wilson, Peter S Hall

**Affiliations:** Edinburgh Cancer Centre, NHS Lothian, Crewe Road South, Edinburgh, UK; Institute of Genetics and Cancer, The University of Edinburgh, Western General Hospital, Edinburgh, UK; Edinburgh Cancer Centre, NHS Lothian, Crewe Road South, Edinburgh, UK; Institute of Genetics and Cancer, The University of Edinburgh, Western General Hospital, Edinburgh, UK; Edinburgh Cancer Centre, NHS Lothian, Crewe Road South, Edinburgh, UK; Institute of Genetics and Cancer, The University of Edinburgh, Western General Hospital, Edinburgh, UK; Edinburgh Cancer Centre, NHS Lothian, Crewe Road South, Edinburgh, UK; The University of Edinburgh Medical School, The University of Edinburgh, Chancellor’s Building, Edinburgh BioQuarter, Edinburgh, UK; Edinburgh Cancer Centre, NHS Lothian, Crewe Road South, Edinburgh, UK; Edinburgh Cancer Centre, NHS Lothian, Crewe Road South, Edinburgh, UK; Institute of Genetics and Cancer, The University of Edinburgh, Western General Hospital, Edinburgh, UK; Institute of Genetics and Cancer, The University of Edinburgh, Western General Hospital, Edinburgh, UK; Usher Institute, Centre for Population Health Sciences, Old Medical School, Teviot Place, Edinburgh, UK; Department of Immunology, Ninewells Hospital and Dundee Medical School, Dundee, UK; Edinburgh Cancer Centre, NHS Lothian, Crewe Road South, Edinburgh, UK; Institute of Genetics and Cancer, The University of Edinburgh, Western General Hospital, Edinburgh, UK; The University of Edinburgh Medical School, The University of Edinburgh, Chancellor’s Building, Edinburgh BioQuarter, Edinburgh, UK; Clinical Infection Research Group, Regional Infectious Diseases Unit, Western General Hospital, Edinburgh, UK; Institute of Genetics and Cancer, The University of Edinburgh, Western General Hospital, Edinburgh, UK; Edinburgh Cancer Centre, NHS Lothian, Crewe Road South, Edinburgh, UK; St John’s Hospital, NHS Lothian, Howden, Livingston, UK; Edinburgh Cancer Centre, NHS Lothian, Crewe Road South, Edinburgh, UK; Institute of Genetics and Cancer, The University of Edinburgh, Western General Hospital, Edinburgh, UK; Clinical Infection Research Group, Regional Infectious Diseases Unit, Western General Hospital, Edinburgh, UK; Edinburgh Cancer Centre, NHS Lothian, Crewe Road South, Edinburgh, UK; Institute of Genetics and Cancer, The University of Edinburgh, Western General Hospital, Edinburgh, UK; Edinburgh Cancer Centre, NHS Lothian, Crewe Road South, Edinburgh, UK; Institute of Genetics and Cancer, The University of Edinburgh, Western General Hospital, Edinburgh, UK

**Keywords:** SARS-CoV-2, COVID-19, SCCAMP, anti-cancer treatment, chemotherapy

## Abstract

**Background:**

Cancer and anti-cancer treatment (ACT) may be risk factors for severe SARS-CoV-2 infection and limited vaccine efficacy. Long–term longitudinal studies are needed to evaluate these risks. The Scottish COVID cancer immunity prevalence (SCCAMP) study characterizes the incidence and outcomes of SARS-CoV-2 infection and vaccination in patients with solid tumors undergoing ACT. This preliminary analysis includes 766 patients recruited since May 2020.

**Methods:**

Patients with solid-organ cancers attending secondary care for active ACT consented to the collection of routine electronic health record data and serial blood samples over 12 months. Blood samples were tested for total SARS-CoV-2 antibody.

**Results:**

A total of 766 participants were recruited between May 28, 2020 and October 31, 2021. Most received cytotoxic chemotherapy (79%). Among the participants, 48 (6.3%) were tested positive for SARS-CoV-2 by PCR. Infection rates were unaffected by ACT, largely aligning with the local population. Mortality proportion was not higher with a recent positive SARS-CoV-2 PCR (10.4% vs 10.6%). Multivariate analysis revealed lower infection rates in vaccinated patients regardless of chemotherapy (HR 0.307 [95% CI, 0.144-0.6548]) or immunotherapy (HR 0.314 [95% CI, 0.041-2.367]) treatment. A total of 96.3% of patients successfully raised SARS-CoV-2 antibodies after >2 vaccines. This was independent of the treatment type.

**Conclusion:**

This is the largest on-going longitudinal real-world dataset of patients undergoing ACT during the early stages of the COVID-19 pandemic. This preliminary analysis demonstrates that patients with solid tumors undergoing ACT have high protection from SARS-CoV-2 infection following COVID-19 vaccination. The SCCAMP study will evaluate long–term COVID-19 antibody trends, focusing on specific ACTs and patient subgroups.

## Teaser

This article characterizes the incidence and outcomes of SARS-CoV-2 infection and vaccination in patients with solid tumors undergoing anti-cancer treatment.

**Implications for Practice**

This is the largest longitudinal study of patients with solid tumors undergoing anti-cancer treatment (ACT—defined here as systemic anti-cancer therapy [SACT] and radiotherapy) during the early stages of the COVID-19 pandemic. We show rates of SARS-CoV-2 infection in patients with cancer mirrored those of the local population, that vaccination was effective, and that treatment type did not impact the rate of SARS-CoV-2 antibody response. Cancer teams should continue to treat patients with cancer during the COVID-19 pandemic with the appropriate ACT and encourage patients to receive a COVID-19 vaccination to maximize their protection from SARS-CoV-2 infection.

## Introduction

Over half-a-billion people across the world have been infected with SARS-CoV-2 (October 11, 2022).^1^ Cancer and systemic anti-cancer treatment (SACT) were identified early as risk factors for infection and related severe illness, citing evidence from previous infection outbreaks.^2-4^ A combination of strategies was deployed to protect patients with cancer, including shielding, minimizing face to face contact, and rationalizing treatment regimens.^5^ Since then, extensive registry data has highlighted that patients with cancer are at increased risk of mortality from SARS-CoV-2 infection.^6,7^ Patients with hematological cancers are at higher risk than solid-organ cancers of severe SARS-CoV-2 illness, and so evaluating these groups separately is important in understanding the risks posed by SARS-CoV-2 infection.^8,9^

There is concern that immunosuppressive therapy, including SACT, may increase COVID-19-related mortality. Studies in solid-organ cancers have identified factors associated with higher COVID-19 mortality, but these have not identified SACT as being among them.^5-7,10^ More recent studies have sought to characterize the immunological response to COVID-19 infection or immunization in patients with cancer undergoing SACT with varying results. A study capturing data from February to May, 2021, including 97 patients with solid-organ cancers and SARS-CoV-2 infection, suggested 89% of patients seroconverted in a cohort where 81% of patients had undergone SACT in the preceding 12 weeks.^11^ Furthermore, vaccination was associated with seroconversion rates of 85% after 2 doses, notably lower than the general population.^12,13^ The vaccination against COVID in cancer (VOICE) trial, conducted over a similar period, showed non-inferiority of 28-days post–vaccination antibody response in a large study comparing patients with solid–organ cancers receiving SACT and individuals without cancer while noting there may be a small cohort who require a further third vaccination.^14^ A recent systematic review of seroconversion following vaccination reported a mean seroconversion rate of 89% (range 63%-99.3%, *n* = 1548 from 13 studies including patients with solid-organ tumors).^15^ These and other data suggest that patients with solid-organ cancers do broadly develop an immune response to SARS-CoV-2, although it may be slightly reduced.^11,12,16^ Follow-up data of patients with solid–organ cancers who received a third vaccine dose suggest both previous vaccine responders and variant non-responders have a marked antibody response that significantly exceeds that seen in patients with hematological malignancies.^17-19^ Other studies estimate an even lower, delayed or waning immune response in this group of patients; significant heterogeneity in study design including an interval of antibody testing, the inclusion of patients with hematological malignancy, follow-up duration, and rate of current treatment with SACT may contribute to the broad range of observed seroconversion.^15,16,20-23^ Real-world, longitudinal data is still needed. Given the importance of maintaining anti-cancer care in an age where SARS-CoV-2 is endemic, it is important to understand the immune response to both SARS-CoV-2 infection and COVID-19 vaccination in patients being treated for cancer.

The primary aims of the Scottish COVID cancer immunity prevalence (SCCAMP) study are to identify the incidence and prevalence of SARS-CoV-2 infection and antibody response in patients with solid–tumor cancers undergoing active anti-cancer treatment (SACT or radiotherapy). The secondary aims are to understand the impact of the previous infection, COVID-19 vaccination, cancer treatment modality, and other patient factors on the presence and duration of this immune response.

In this report, we describe outcomes in a cohort of patients receiving active cancer treatment during the COVID-19 pandemic between May, 2020 and October, 2021 who have contributed serial blood samples for antibody testing when attending for treatment.

## Materials and Methods

### Study Design and Data Collation

SCCAMP is a prospective observational study, and the SCCAMP study protocol is available at: https://cancer-data.ecrc.ed.ac.uk/projects/sccamp/sccamp-information-for-professionals/. See Supplemental Methods for additional source information. In brief, patients were eligible if they were over the age of 18 with a confirmed diagnosis of solid–organ cancer and/or received cancer treatment in the last 12 months. Consent was provided when attending for ACT, primarily SACT, at the Edinburgh Cancer Centre (ECC) either the Western General Hospital (WGH), Edinburgh or St John’s Hospital (SJH), Livingston (NHS Lothian NRS BioResource, BioBank SR1418, NHS Research Ethics Committee [REC]: 20/ES/0061 and SCCAMP, NHS Research Ethics Committee [REC] REC: 20/SS/0109). Blood samples were taken for antibody testing at consent up to a maximum of 5 collections up to 1 year from consent (approximately +42 days, +84 days, +6 months, and +1 year). Clinical information was obtained through data linkage from routine NHS Electronic Patient Records, the chemotherapy prescribing system ChemoCare, and Public Health Scotland (PHS). Socioeconomic status was calculated from residential postcodes at recruitment crossed to Scottish index of multiple deprivation (SMID) scores. Quan–Charlson indices (QCIs) for 5 years before recruitment, were calculated using the weightings of Quan et al.^24^ but excluding cancer as comorbidity. Total prescribed medicines within 1 year before consent were also extracted. Patient data were compared to their recruitment date (“baseline date”). Treatment regimens were hierarchically classified into 1 of 3 classes (chemotherapy > immunotherapy > other) for the duration of the study including 6 months before recruitment. Patients receiving more than 1 therapy were classified by their hierarchy [Supplementary Table S1]. All data was up to date as of October 31, 2021, at which point data was censored.

### COVID-19 PCR and Antibody Data

COVID-19-positive cases were defined as cases with supporting positive-PCR test data from PHS. Monthly incident rates and cumulative total calculated for the combined local authorities in which the 2 hospital sites reside (the City of Edinburgh and West Lothian, UK). Population COVID-19 infection rates were adjusted to per 1000 population values and age-corrected to remove individuals under the age of 25 (see Supplemental Methods). Vaccination data within the cancer cohort was provided by PHS.

Serum samples were tested via the validated Siemens Total (IgG/M and IgA) SARS-CoV-2 antibody assay, which is licensed and approved by the EU and FDA, at Ninewells Hospital, NHS Tayside with thresholds for antibody applied as previously defined.^25-27^ See Supplemental Methods for information on analysis.

### Data Visualization and Statistical Analysis

All analysis was carried out using base R version 4.0.5. For all univariate and multivariate analyses, COVID-19-positive cases were only considered if they occurred after the date of the most recent cancer treatment (43/48 cases). Univariate and multivariate analysis was carried out using the Survival package 3.2-13 to compute the Cox proportional hazards regression models.

## Results

### Patient Demographics

**Figure 1.**
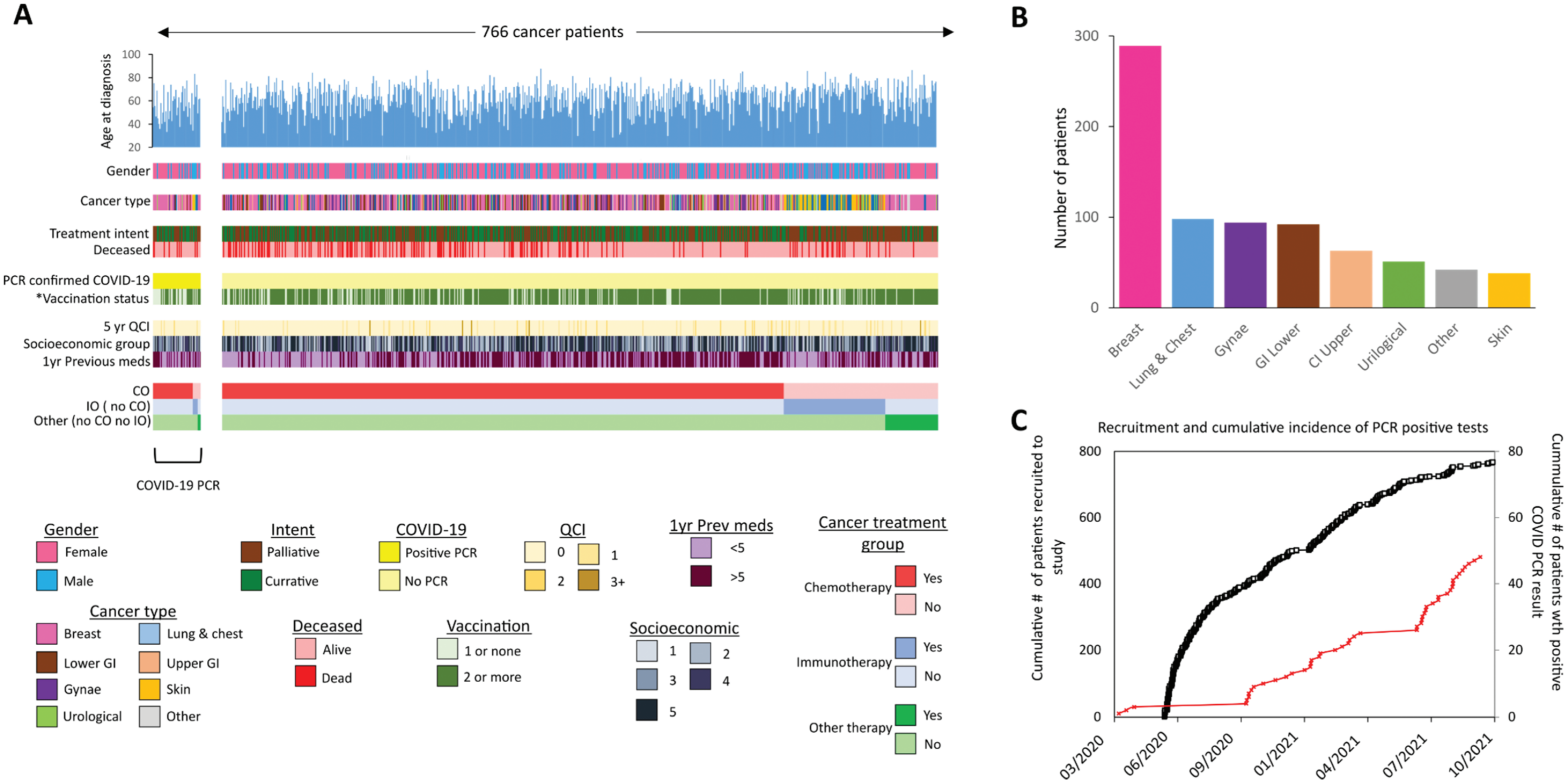
(**A**) Graphical summary of available data in the SCCAMP study across the 767 patients. Patients are stratified by their COVID-19 PCR status (yellow) and then ranked by their treatment type. The color key for each data type is shown below. *Vaccination status is defined as status at time of COVID-19 positive PCR or end of study if not positive (**B**) Plot of patient numbers split by cancer type (**C**) Plot of patient recruitment throughout the study (black line) with confirmed cases of COVID-19 overlaid (red line).

A total of 767 patients attending ACT consented between May 28, 2020 and October 28, 2021, of whom 766 were included for analysis (Supplemental Fig. S1). Patients were recruited across 2 sites within Edinburgh and West Lothian, UK (612, 79.9%, at the WGH in Edinburgh, UK and 154, 20.1%, at St John’s Hospital in Livingstone, UK) with a median follow-up of 405 days (Fig. 1A, 1B, and Table 1).

**Table 1.** Summary of patient data in the SCCAMP study.

Characteristics	*N*	Total cohort (%)
Total cases	766	
Follow-up period, days, median (range)	405 (3-521)	
Centre		
Western General Hospital, Edinburgh, UK	612	79.9
St John’s Hospital, Livingstone	154	20.1
Age at recruitment, years, median (range)	62.8 (26-87.8)	
Gender		
Female	510	66.6
Male	256	33.4
Cancer type		
Breast cancer	289	37.7
Lung and chest	98	12.8
Gynecology	94	12.3
Lower GI	92	12.0
Upper GI	63	8.2
Urological	51	6.7
Skin	38	5.0
Other	41	5.4
Socioeconomic status (SMID quintiles)		
1	82	10.7
2	153	20.0
3	130	17.0
4	129	16.8
5	272	35.5
Comorbidity: Quan–Charlson score ≤5 years		
0	690	90.1
1	51	6.7
2	20	2.6
≥3	5	0.7
Comorbidity: prescribed medications ≤1 year, median (range)	5 (0-28)	
Vaccination status at end of study or at death		
≤1	155	20.2
≥2	611	79.8
PCR confirmed COVID-19		
Yes (<6 months before treatment)	5	0.7
Yes (during treatment)	43	5.6
No	718	93.7
Treatment intent		
Palliative	441	57.6
Curative	325	42.4
Treated with chemotherapy	603	78.7
Treated with immunotherapy; no chemotherapy	107	14.0
Other treatment; no immunotherapy, no chemotherapy	56	7.3
Death details		
Died within 28 days of PCR-confirmed COVID-19	2	0.3 (4.2% COVID-19 positive)
Died within 90 days of PCR-confirmed COVID-19	5	0.7 (10.4% COVID-19 positive)
Died <2 years after PCR-confirmed COVID-19	9	1.9 (18.7% COVID-19 positive)
Died, no PCR-confirmed COVID-19	158	20.6
Alive at end of study	599	78.2

The median age across the cohort was 62.8 years (min. 26, max. 87.8) with 510 females (66.6%), and 256 males (33.4%) (Supplemental Fig. S2 and Table 1). Although the most common cancer types were breast cancer (*n* = 289-37.7%), our cohort included a wide range of cancer types (Fig. 1A, 1B, Supplementary Table 1, and Supplementary Fig. S3). Patients represented a wide range of socio-economic groups, with most in the highest SIMD quintile (Q5; *n* = 272-35.5%) (Supplemental Fig. S4). Five-year comorbidity as described by QCI score defined most patients (*n* = 690; 90.1%) as being without any associated comorbidity (Fig. 1A). QCI scores associated with previous medication (1-year pre-recruitment) were also investigated across the patients revealing a median of 5 previously prescribed medications (range: 0-28).

Overall, 325/766 patients (42.4%) were being treated with curative intent (Supplementary Tables S2 and S3). Of the 441 patients receiving palliative care, 268 (60.8%) had received previous cancer therapy over the past ten years, 62 (14.1%) of whom had received more than 3 treatment regimens (Supplementary Table S3). Across the duration of the study, 603/766 (78.7%) were classified as receiving cytotoxic therapy, 107/766 were classified as receiving immunotherapy (in the absence of cytotoxic therapy; 14%), and 56/766 (7%) were classified as receiving another treatment in the absence of cytotoxic and immunotherapeutic intervention (Fig. 1A and Table 1). A total of 497/766 (64.9%) patients received more than 1 therapeutic intervention type (Supplemental Fig. S5).

### COVID-19 Infection Rates Within the Cancer Cohort

**Figure 2.**
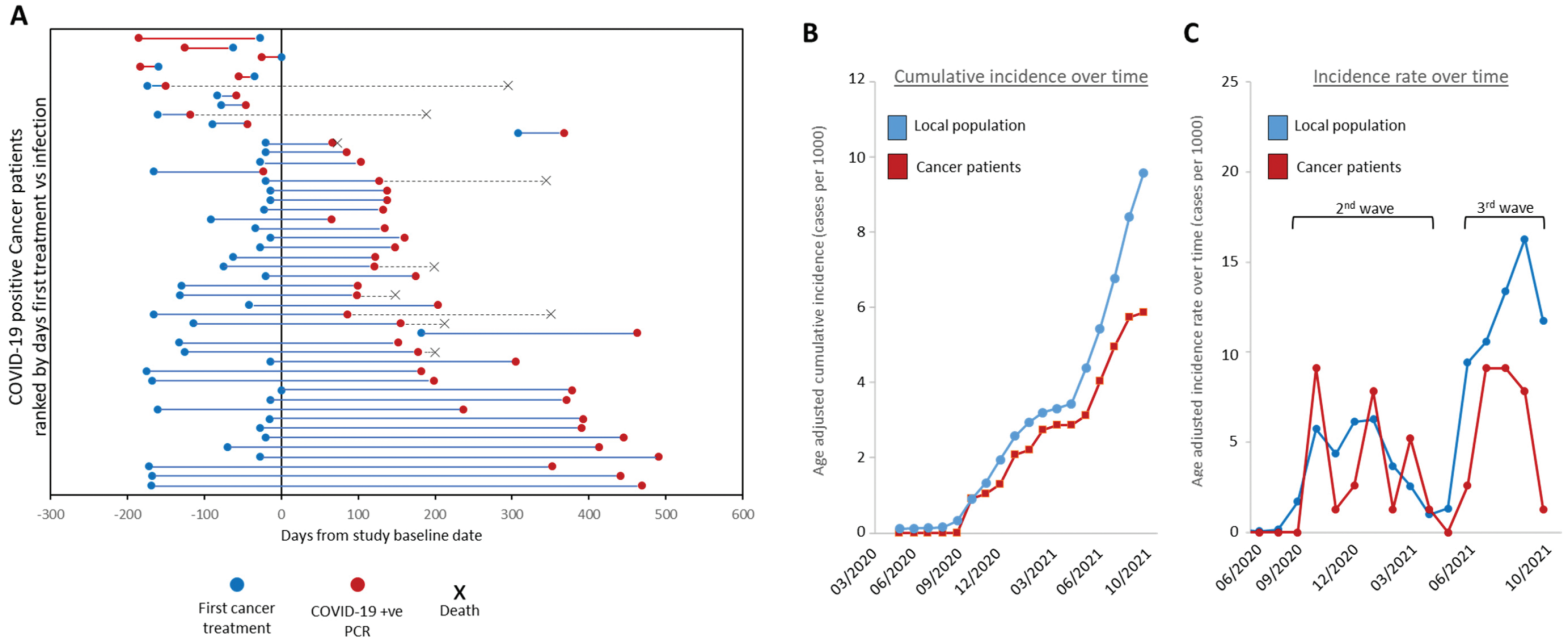
(**A**) Plot of time between first cancer treatment (blue dots), COVID-19 infection (red dots) and death (black cross) across the 48 COVID-19 positive cancer patients in the study ranked by time between treatment and infection. Red bars denote cases where infection occurred before first treatment, blue bars where infection occurs after first treatment. Plots display time with respect to date of recruitment into the study. (**B**) Age adjusted COVID-19 cumulative incidence over time (**C**) incidence rate over time plots for SCCAMP patients (red) and the local general population (blue). Values are presented as PCR confirmed cases per 1000 per month.

Over the study period, 48/766 cancer patients (6.3%) had a recorded positive COVID-19 PCR test. (Figs. 1B, 2A). In total, 5 patients tested positive for COVID-19 before the start of their cancer treatment; however, these individuals went on to receive treatment within 6 months of infection. Excluding these 5 cases, the median time from first cancer treatment to COVID-19 infection was 230 days (min. 2 days, max. 638 days). In 10 of the 48 cases, remained positive in at least 1 follow-up PCR test (median follow-up 7 days) (Supplemental Fig. S6). No cases of reinfection with COVID-19 were seen. Cases were found across 7 of the 8 cancer type groups, although the proportion of cases in each category was not randomly distributed (Chi-squared test = 5.3E−05), with more cases in those with breast cancer (Supplemental Fig. S3). Similar levels of COVID-19 infection were detected across patients split by curative and palliative intent or by the number of recent medications (Supplementary Tables S2, S4).

Comparing COVID rates within the cancer patients against age-adjusted NRS (NRS BioResource) population data over the same geographic area, the cumulative incidence and incidence rate of cases were similar between our cohort and the general population until approximately May, 2021, when the proportion started to increase in the general population (Fig. 2B, 2C). The age-adjusted cumulative incidence in May, 2021 was 3.4 and 2.9 per 1000 for the local general population and the SCCAMP cancer cohort respectively and by the end of study 9.6 per 1000 for the local population and 5.9 per 1000 in the cancer cohort (Fig. 2B, 2C).

### Mortality and Association of Cancer Treatment with COVID-19 Infection

Across the study period, 2/48 patients (4.16%) died within 28 days of a positive PCR result (Supplemental Fig. S7). When this was expanded to include all deaths within 90 days or across the study period, this resulted in 5 (10.4%) and 9 (18.7%, median survival days from recruitment 200) deaths, respectively. A total of 8/9 (88.9%) of those who died at any time after a COVID-19 infection were being treated with palliative intent. By contrast, 158 cancer patients who did not report a COVID-19 positive PCR result died over the entirety of the study period (20.6% total cohort, median survival days from recruitment 192), 86.8% of whom were being treated with palliative intent (Table 1).

Median time from treatment initiation to COVID-19 infection was 196 days across these 9 patients (min. 23 days, max. 304 days) compared with 246 days for patients who were alive at the end of the study (excluding cases where COVID-19 was contracted before treatment initiation), although this did not reach significance (2-tailed *t*-test, *P*-value = .079) (Fig. 2A and Supplemental Fig. S8).

Those in the cohort who had a positive SARS-Cov-2 PCR result during the study period were younger than those who had not (Supplemental Fig. S2), although there was no significant difference between the ages of all cancer patients who died during the study to those who died either at any time after COVID-19 infection, or within 28 days (Supplemental Fig. S2). Interestingly, despite maintaining a similar patient cohort over time (including proportions split by age, gender, curative intent, and comorbidity), patients who had experienced a COVID-19 infection who died within the first half of the study exhibited significantly shorter times between infection to death than those in the second half of the study (First 9 months: *n* = 4, May, 2020 to January, 2021, median days from infection to death = 40; second 9 months: *n* = 5, February, 2021 to October, 2021, median days from infection to death = 242; 2-tailed *t*-test *P*-value = .023).

There was no significant difference in SARS-CoV-2 infection rates depending on the treatment type received. Excluding cases where COVID-19 was contracted before treatment initiation (*n* = 5), positive COVID-19 PCR rates between patients in the chemotherapy treatment group were 6% (36/599), 3.8% in the immunotherapy group (4/106), and 5.4% (3/56) in those who received other treatments (Supplementary Table S4). In adjusted models for COVID-19-free days, there was no significant difference in positive COVID-19 PCR rates by treatment group (chemotherapy: hazard ratio [HR] 1.41 [95% CI 0.63-3.18]; immunotherapy: 0.62 [95% CI 0.22-1.74]; other treatments: HR 0.94 [95% CI 0.29-3.02]; chemotherapy without immunotherapy: HR 1.71 [95% CI 0.80-3.72]).

In comparison to those who did not have a positive COVID-19 PCR result, patients who tested positive for COVID-19 (PCR) were younger and less likely to be double vaccinated (>60 years age: 58.3%, no COVID-19 vs 45.9% COVID-19+ve, 12.3% decrease in COVID-19 infected patients; [double vaccination: 79.1%, no COVID-19 vs 59.5% COVID-19+ve, 19.7% decrease in COVID-19 infected patients]). There was a less than 10% difference between the percentage of COVID-19 PCR-positive patients and the remaining cohort in the following factors: QCI scores, numbers of previous medications, socioeconomic status scores, cancer treatment class, and gender (Table 2).

**Table 2.** Patient characteristics by those who tested positive for COVID-19 and those who did not, with difference (%) between these groups relative to (statistical test: 2 proportion *Z*-test, *P*-values shown).

	COVID-19 positive patients after treatment *n* = 43 (%)	Non-COVID-19 positive patients *n* = 718	COVID-19 positive patients (∆%)	2 proportion	
				*Z*-test	*P*-value
Age >60, years	19 (44.2)	419 (58.4)	−14.20	.067	<0.1*
Gender: female	30 (69.8)	476 (66.3)	3.50	.627	
Socioeconomic high	20 (46.5)	379 (52.8)	−6.30	.421	
QCI comorbidity	3 (7.0)	62 (8.6)	−1.70	.714	
1-year medications >5	20 (46.5)	341 (47.5)	−0.98	.896	
Chemotherapy group	36 (83.7)	562 (78.2)	5.45	.394	
Immunotherapy group	4 (9.3)	102 (14.2)	−4.90	.367	
Other group	3 (7)	53 (7.4)	−0.40	.922	
Vaccine: 2>	22 (51.2)	569 (79.2)	−28.10	1.87E-05	<0.05***

Abbreviation: QCI, Quan–Charlson indices.

Multivariate analysis adjusting for age, gender, QCI scores, previous medications, and socioeconomic factors reveals that vaccination reduced the risk of having a positive COVID-19 PCR test across the entire patient cohort (HR 0.26 [95% CI, 0.14-0.48]) (Table 3 and Supplemental Methods). This was regardless of treatment by either chemotherapy only or immunotherapy only; with hazard ratios of 0.21 (95%, CI, 0.10-0.41) or 0.314 (95% CI, 0.041-2.37), respectively.

**Table 3.** Multivariate analysis to determine the risk of having a positive COVID-19 PCR test across patients in the SCCAMP study.

	Hazard ratio	Lower 0.95	Upper 0.95	*P*-value	Significance
Age: >60 years	0.59	0.31	1.09	.0933	*
1 year previous medication: high	0.93	0.51	1.72	.8261	
Socio-economic: high	0.94	0.51	1.74	.8442	
5 years comorbidity QCI > 0	0.58	0.18	1.89	.3645	
Gender: female	1.23	0.63	2.41	.5376	
Vaccinated	0.26	0.14	0.48	1.50E-05	***

Summary of the hazard ratios, confidence intervals of the hazard ratios as lower 95% bound and upper 95% bound.

*P*-value and significance: ***, .001; **, .01; and *, .05; 0.1 across clinical variables age (>60 years).

Recent previous medications (1-year medicines >5).

High socioeconomic score = SMID quintiles ≥4.

High medication comorbidity = prescribed medications in 1 year prior to recruitment >5, comorbidity QCI (Quan–Charlson indices) >0 in 5 years prior to recruitment, gender = Female and vaccination status (at least 2 doses). For full threshold information see Supplemental Methods.

### COVID-19 Vaccination in the Cancer Cohort

**Figure 3.**
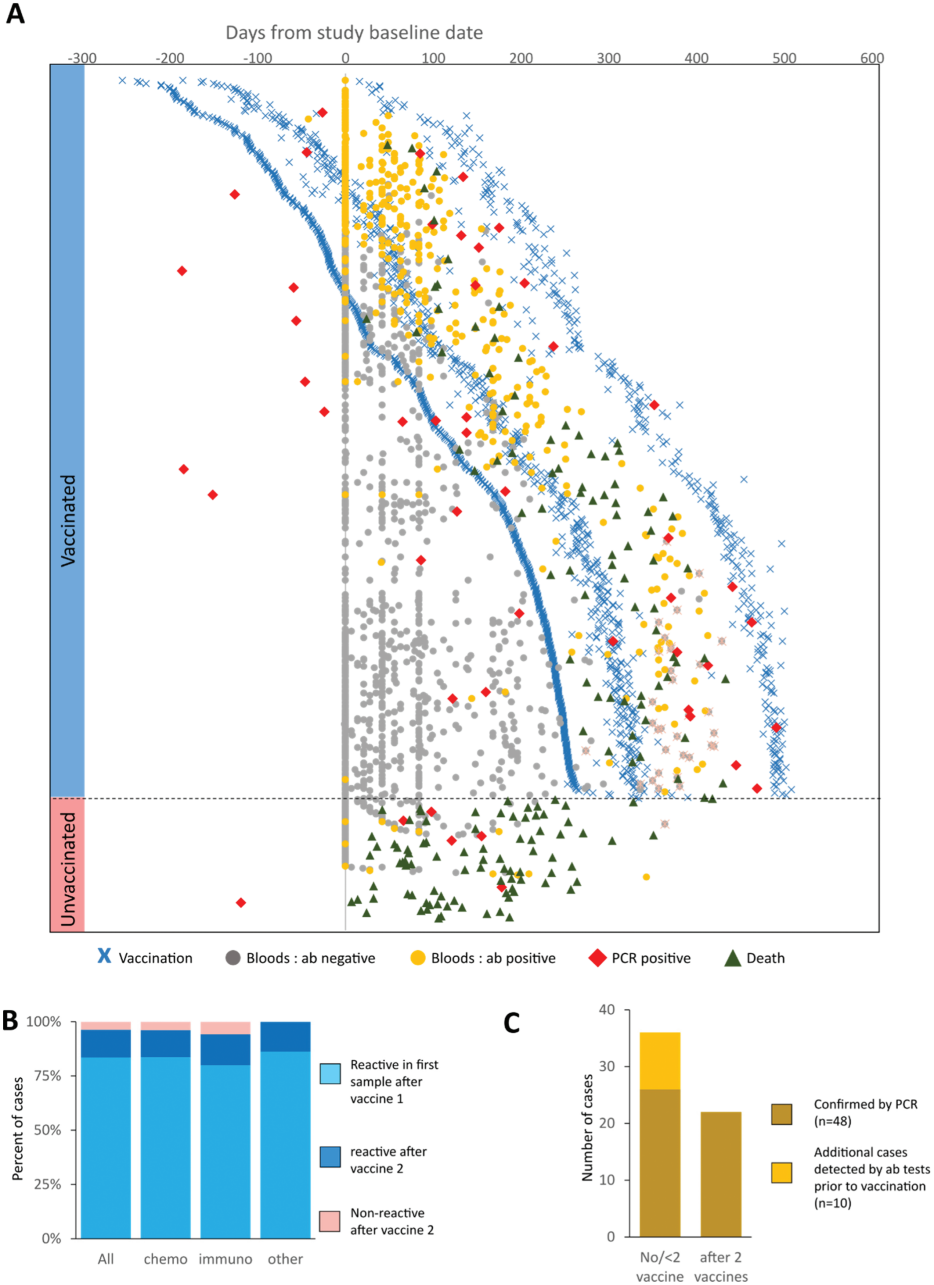
(**A**) Plot of timings of vaccinations, antibody data points, and PCR test results for the 766 patients with respect to baseline dates. Patients are arranged in order of their first vaccination date. Vaccinations are denoted by crosses (light blue = 1st, dark blue = 2nd, and green = 3rd). Dots denote antibody test collection dates w.r.t. Baseline data. Grey dots = antibody non-reactive result, yellow dots = antibody reactive result, red dot = date of PCR positive test. Blue bar on the left denotes patients who had received at least 1 vaccine dose, the pink bar at the bottom left denotes unvaccinated patients. Dashed line separates these 2 groups (**B**) Stacked bar plot of percentage of patients reporting antibody reactivity split by response type. Data is split for all available patient data as well as by treatment class. Light blue = reactive in first sample after vaccine 1, dark blue = reactive after vaccine 2, pink = non-reactive after vaccine 2. Data shows samples for which there is either a reactive antibody result in the first sample after vaccine 1 and/or antibody data available >14 days after second vaccination (**C**) Plot of number of COVID-19 cases confirmed by PCR test (gold) or suspected infection through antibody reactivity before vaccination, split by patient vaccination status. GI = Gastrointestinal.

The COVID-19 vaccination program began in Scotland on December 8, 2020, with patients with cancer among those prioritized. A total of 730/766 patients from our cohort were alive when the vaccination program began (95.3%). By the date of data censoring, 155 patients received less than 2 vaccinations (20.2% of the cohort). Among 117 unvaccinated patients, 59% died; 32% died before the program began (*n* = 37) and 27% died within 6 months of the first vaccine (*n* = 32). By contrast, 246 had received 2 vaccine doses (32.1%) and 365 had received 2 doses plus a booster (47.7%) (Supplemental Fig. S9 and Fig. 3A). As such 79.8% of the cancer cohort received at least 2 vaccine doses over this period. This compares to vaccination rates of 71.5% for 2 vaccines and 13.2% for <2 vaccines across the national population of Scotland over the same time period.^28^ Most patients in the study received AstraZeneca vaccines either as their first (88.2%) or second (87.6%) vaccine with a minority receiving Pfizer as their first (11.7%) or second (12.1%) dose. By contrast, most booster vaccines were either Pfizer (65.2%) or Moderna (34.5%) (Supplemental Fig. S9A).

Proportions of deaths from all causes differed across these 3 groups with a death recorded for 118/155 non-fully vaccinated patients (76.1%), 49/256 double-dosed patients (20%) and no deaths recorded for triple-dosed patients. Notably, the only cohort in which COVID-19-related deaths were reported (classified here as a death recorded <90 days after positive PCR) were unvaccinated individuals (*n* = 5), 2 of which were within 28 days (Supplemental Fig. S9B].

### Antibody Positive Over Time and Cumulative Incidence

We assessed antibody response to vaccination and undetected COVID-19 infection events. At the time of analysis, antibody data for at least 1-time point was available for 591 patients (77.1% of cohort), with a total of 1418 samples collected longitudinally at baseline and then at 1.5, 3, 6, and 12 month follow-up periods. Across these 591 patients, 348 (45.4%) contained data that overlapped with time points at least 14 days after the date of the first vaccination (Fig. 3A). In total, we collected a median of 2 (min. 1, max. 5) antibody samples per patient over a median span of 95 days (min. 14 days, max. 429 days) (Supplemental Fig. S10). At least 1 reactive antibody result was noted in 304/589 patients (51.6%) which was restricted to 285/347 patients (82.1%) for which antibody data was at least 14 days after vaccination 1.

A total of 297 patients fulfilled our criteria with which we could calculate seroconversion rates (see Supplemental Methods). A total of 248/297 (83.5%) patients displayed a reactive result in the first blood sample after vaccination 1 (Fig. 3B) and 38/297 cases displayed an initial non-reactive result >14 days after their first vaccination before becoming reactive at a later stage (12.8%) (7/38 pre-second vaccination, 31/38 post-second vaccination), with only 11 patients returning no positive results >14 days post-second vaccination (Supplemental Figs. S11 and S12). Overall, we observed an antibody response rate to vaccination of 96.3%. No differences were observed when stratifying these response classes by treatment type (Fig. 3C), nor by vaccination manufacturer (Supplemental Fig. S13).

Across all samples, we observed only 4 cases where seroconversion was subsequently lost; 3 of whom had received 2 doses of the vaccine at the time of reversion (median time to reversion at 42 days) and 1 case who reverted before their first vaccination (asymptomatic infection) (Supplemental Fig. S14).

We noted a number of additional COVID-19 infections, before any vaccination, which was not detected by PCR tests (*n* = 10) (Fig. 3A). Combining these additional pre-vaccination cases with PCR-confirmed COVID-19 cases results in 36 positive cases in patients before a 2nd vaccination (26 PCR positive, 10 additional pre-vaccination antibody reactive) compared to 22 PCR-positive cases in patients after their 2nd vaccination (Fig. 3C].

## Discussion

We describe the findings of the SCCAMP study which seeks to characterize the pattern of SARS-CoV-2 infection and vaccination immune response in a cohort of patients with solid tumors undergoing anti-cancer treatment between May, 2020 and October, 2021. This period represents the earliest stages of the COVID-19 pandemic, and includes the second and third waves, driven by the alpha, and delta variants of concern (VOC), respectively.

Studies have previously highlighted a higher mortality risk for patients with cancer, and proposed that certain groups of patients are at higher risk of severe SARS-CoV-2 infection, such as those with advanced disease or lung cancer.^5-7,29,30^ Many early studies could be influenced by a highly diverse definition of cancer and, importantly, changes in anti-cancer treatment policies during the pandemic.^5^ More recent studies have evaluated the immune response of patients with cancer following SARS-CoV-2 infection and vaccination, with a wide variation in proposed responses owing to differences in study populations.^11,12,14,16,20,21^ Furthermore, studies evaluating booster vaccinations and longer-term response highlight the evolving questions around the longevity of response, particularly concerning new SARS-CoV-2 variants.^17,18,23^ In contrast with much of the UK, the South East Scotland Cancer Network (SCAN), which comprises both hospitals reported here, had largely normalized SACT attendance rates by June/July, 2020 (vs −31.2% in England and Northern Ireland).^31,32^ SCCAMP therefore offers an opportunity to understand trends in SARS-CoV-2 infection and immunity in a cohort of cancer patients similar to that of a pre-COVID-19 era, and during a period of multiple waves of SARS-CoV-2 variants. We report trends in COVID-19 incidence in this population, in addition to immunity patterns, in patients who are deemed well enough to undergo anti-cancer treatment, are outpatients, and were asymptomatic at the time of sampling. This is important given the likelihood of ongoing waves of SARS-CoV-2, and the need to continue anti-cancer treatment to avoid the risk of cancer mortality exceeding that of SARS-CoV-2.

Our data demonstrate that an actively treated cohort of cancer patients had a similar incidence of SARS-CoV-2 to the regional population. The small number of cases in our cohort makes it difficult to make inferences about overall risk, but we did not observe a higher risk depending on treatment type, in keeping with other studies.^6,7^ The mortality proportion between those who ever and never had a positive COVID-19 PCR test were broadly similar, although mortality from SARS-CoV-2 infection was notably lower than in previous observational studies.^5-7^ This likely reflects the relative fitness of this cohort, given that all patients recruited here were asymptomatic on the days they gave samples, were outpatients, had few comorbidities, and were deemed fit enough for ACT. In patients who tested positive for SARS-CoV-2 and died during the follow-up period, the time between SARS-Cov-2 infection and death was shorter in those diagnosed during the first half of the study. We did not observe any changes in the proportion of patient characteristics (age, treatment intent, COVID-19 infection rates, previous medications), which suggests this change was driven by factors such as vaccination, changes in patient exposure (ie, lockdown easing, and in-person appointments), improvements to COVID-19 management, access to early COVID-19 testing, and possibly COVID-19 variants. We had relatively few patients with lung cancer, a group considered at higher risk from SARS-CoV-2,^10^ which may also have influenced this result. Overall, the low number of deaths means it is important not to over-interpret death data.

After approximately May, 2021, the rates of SARS-CoV-2 infection in the regional population were higher than in the SCCAMP cohort, suggesting a protective effect from vaccination given that patients with cancer were prioritized. Despite our small rate of SARS-CoV-2 infection, we still observed a significant reduction in the number of positive COVID-19 PCR results in patients who had received 2 or more doses of any COVID-19 vaccination. These data highlight that patients undergoing active treatment for cancer gain significant protection from SARS-CoV-2 infection by receiving a COVID-19 vaccination.

We found that nearly 50% of patients had already received a booster COVID-19 vaccination at the time of censoring (October 31) in comparison with the Scottish rate (13.2% – scot.gov), and an overall higher proportion of our cohort had received at least 2 doses than the general population at the same time. The proportion of patients (20.3%) that had received no or a single vaccine is likely reflective of patients who died of their cancer or related causes before receiving both doses. Our data suggest that most patients with solid tumors who are receiving ACT are both being reached and are engaged with COVID-19 vaccination.

We observed that 96.3% of patients in our cohort who were vaccinated had seroconverted when considering any positive result post-first vaccination, and all results >14 days post-second vaccination as the denominator. This is higher than reported in some studies^12,20,21^ and is comparable with a large study comparing seroconversion at 28 days post-second vaccination.^14^ Importantly, there was no difference in response between patients receiving chemotherapy, immunotherapy, or other treatments, which builds on similar observations noted in the VOICE study.^14^ Others have reported that patients with cancer may have delayed seroconversion or earlier waning of the antibody response.^16,21,23^ Booster vaccination has been shown to elicit an antibody response both in previous vaccination responders and non-responders, including in a cohort of patients with exclusively metastatic disease.^17-19,33,34^ The timing of boosters, including around treatment, represents an ongoing area for uncertainty.^34^ Previously reported cohorts targeted patients with confirmed SARS-CoV-2 infection over a discrete period (both in terms of recruitment and antibody testing strategy).^11,12,14^ We may have been able to capture this with our longer follow-up and denominator definition. Our longitudinal, rolling recruitment strategy aimed to cover a broad church of patients, and therefore, may more accurately represent the cancer population as a whole. This will be an area of subsequent study as SCCAMP continues to evaluate the serological response of patients over time since vaccination, particularly as it represents, to our knowledge, the largest ongoing repository of real-world patient samples.

We note some limitations of SCCAMP. Our study has not evaluated specific aspects of the immune response, including T-cell response or quantitative, longitudinal assessment of different antibody classes. This may reveal a differential in the longevity or robustness of the immune response to SARS-CoV-2 in patients treated with SACT. Furthermore, we cannot comment on the incidence of SARS-CoV-2 variants as this data was not available through PHS or electronic patient records. Given the small number of natural infections and high rates of seroconversion, meaningful interpretation of the prevalence or durability of immune response between variants would be limited. As regular asymptomatic PCR screening was not routine clinical practice during the studied period, asymptomatic cases, particularly in the post-vaccination period, will likely be underestimated. However, broadly we can still presume that our results are reflective of symptomatic infection, and our comments regarding COVID-19 PCR test results should be interpreted accordingly. As noted where relevant in this analysis, the number of patients with a positive SARS-CoV-2 PCR were relatively small, and consequently, we have been cautious in over-analyzing sub-categories of this group. Our real-world follow-up strategy inevitably results in not all patients providing all or as many samples for antibody testing. This is also reflective of the need to balance between exposing patients to contact only when needed and the dynamic research changes demanded by the waves of SARS-CoV-2 infection. Our study relies on publicly available and published data to provide control data for a non-cancer population, and although patients on other treatments not thought to significantly impact the immune system (“other”) have acted as a control group for our cohort, we acknowledge that some treatments in this category (eg, targeted therapies) can impact on the immune system.

Subsequent analysis this cohort will include the trend of COVID-19 antibodies over time, the effect of booster vaccinations and ACT, and look at specific subgroups to explore the immune profile in greater detail. With current recommendations suggesting the fourth dose of vaccine for patients undergoing treatment with immunosuppressive SACT, it is a topic of an ongoing investigation to uncover the dynamics of the immune response in this population which may help to inform decisions about the timing of boosters around treatment.^35-37^

## Conclusion

This preliminary report from SCCAMP suggests that in patients with solid tumors receiving ACT during the early stages of the COVID-19 pandemic, symptomatic SARS-CoV-2 infection rates have been comparable to the general population. Significant protection is offered by vaccination, both in terms of antibody response and survival, and irrespective of the type of ACT received. Vaccination against COVID-19 should be widely encouraged in patients with cancer undergoing treatment.

## Supplementary Material

oyac257_suppl_Supplementary_Material

## Data Availability

Publicly available COVID-19 data from Public Health Scotland (PHS) was used where indicated in this study. The collated routine clinical data analyzed in this study resided within NHS databases and is not available for open access on the basis of patient confidentiality. Anonymized data may be available for studies where ethical approval has been obtained, and the authors should be contacted directly regarding this.

## Appendix. SCCAMP Advisory Group

In addition to the authors listed in the byline, the advisory group included the following people: Mark Baxter and Russell Petty: Dundee Cancer Centre, Ninewells Hospital and Dundee Medical School, Dundee, UK; Paul Brennan—Centre for Regenerative Medicine and Cancer Research UK Edinburgh Centre, Institute for Regeneration and Repair, The University of Edinburgh, Edinburgh, UK; Ruth Fullerton, Duncan McLaren, Iain Phillips, Paul Ramage, and Stefan Symeonides: Edinburgh Cancer Centre, Edinburgh, UK; Margot Watson: Patient Representative Group
