# Supplementary material for: The Scottish COVID Cancer Immunity Prevalence Study: A Longitudinal Study of SARS-CoV-2 Immune Response in Patients Receiving Anti–Cancer Treatment
Source: Oncologist. 2023 Jan 31;28(3):e145–55. doi: 10.1093/oncolo/oyac257 (PMC10020811; doi:10.1093/oncolo/oyac257)
Supplement: oyac257_suppl_Supplementary_Material [file oyac257_suppl_supplementary_material.docx]

**Supplemental data: The Scottish COVID CAncer iMmunity Prevalence (SCCAMP) Study: A longitudinal study of SARS-CoV-2 immune response in patients receiving anti-cancer treatment**

Karin Purshouse^1,2^*, John P Thomson^1,2^*, Mahéva Vallet^1,2^ *, Lorna Alexander^1^, Isaac Bonisteel^3^, Maree Brennan^1^, David A Cameron^1,2^, Jonine D Figueroa^2^ ^,4^, Elizabeth Furrie^5^, Pamela Haig^1,2^, Mattea Heck^3^, Hugh McCaughan^6^, Paul Mitchell^2^, Heather McVicars^1^, Lorraine Primrose^7^, Kate Templeton^6^, Natalie Wilson^1,2^, Peter S Hall^1,2^+

*Co-lead author

+ Corresponding author

Affiliations:

1. Edinburgh Cancer Centre, NHS Lothian, Crewe Road South, Edinburgh EH4 2XU, UK

2. Institute of Genetics and Cancer, The University of Edinburgh, Western General Hospital, Crewe Road, Edinburgh EH4 2XU

3. The University of Edinburgh Medical School, The Chancellor’s Building, Edinburgh BioQuarter, 49 Little France Crescent, Edinburgh, EH16 4SB

4. Usher Institute, Centre for Population Health Sciences, Old Medical School, Teviot Place, Edinburgh, EH8 9AG

5. Department of Immunology, Ninewells Hospital and Dundee Medical School, Dundee, DD1 9SY, UK

6. Clinical Infection Research Group, Regional Infectious Diseases Unit, Western General Hospital, Edinburgh, UK

7. St John’s Hospital, NHS Lothian, Howden Road West, Howden, Livingston EH54 6PP

**Supplemental Methods** Page 2

**Supplemental Figures** Page 5

**Supplemental Tables** Page 18

**Supplemental methods**

Full Study Design

The SCCAMP study protocol is available on <https://cancer-data.ecrc.ed.ac.uk/projects/sccamp/sccamp-information-for-professionals/> . Patients were eligible if they were over the age of 18 with a confirmed diagnosis of solid organ cancer, defined as cancer or metastasis in situ, and/or receiving cancer treatment (surgery, radiotherapy, hormone therapy, chemotherapy, targeted therapy, immunotherapy) in the last 12 months, and attending for outpatient Cancer Centre Care. Patients were not eligible if they had a concurrent haematological malignancy due to the different clinical profile of this cohort.

Patients consented to a Biobank (NHS Lothian NRS BioResource, BioBank SR1418, NHS Research Ethics Committee (REC):20/ES/0061) or the SCCAMP study (REC:20/ES/0076) when attending for anti-cancer treatment (ACT), primarily SACT, at the Edinburgh Cancer Centre (ECC) either at the Western General Hospital (WGH), Edinburgh or St John’s Hospital (SJH), Livingston, providing blood samples and consenting to anonymised review of routine clinical data.

Patients provided further blood samples up to a maximum of five over 1 year from consent (approx. +42 days, +84 days, +6 months, +1 year), when returning for further routine out-patient care.  Patients were recruited throughout the period (Figure 1B) alongside follow-up sample acquisition. Although the protocol permitted patients to be recalled to provide samples as study visits, we prioritised fitting in samples with routine out-patient care to minimise additional visits for patients and consequently potential contact which might expose them to SARS-Cov-2 infection. Serum samples were tested via the validated Siemens Total (IgG/M and IgA) SARS-Cov-2 antibody assay at Ninewells Hospital, NHS Tayside (19-21).

Data collation – source links:

Clinical information was obtained through data linkage from routine Electronic Patient Records including prescribing systems (ChemoCare™ - <https://www.scan.scot.nhs.uk/projects/chemocare/>), PCR/vaccine data was obtained from Public Health Scotland (<https://www.publichealthscotland.scot/>), and comorbidity data was obtained from SMR01 (General/Acute Inpatient and Day Case - https://www.ndc.scot.nhs.uk/Data-Dictionary/SMR-Datasets/SMR01-General-Acute-Inpatient-and-Day-Case/) and Prescribing Information System (PIS - https://www.ndc.scot.nhs.uk/National-Datasets/data.asp?SubID=9)

Data collation – cancer type stratification:

Cancer type at recruitment was extracted and stratified into one of 8 groups based on the most dominant cancer types seen in the study as follows: breast, lung and chest, gynae, lower gastrointestinal (GI), upper GI, urological, Skin or other. “Other” cancer types include: head and neck (n=16), soft tissue sarcoma (n=8), cancer of unknown primary (n=8), prostate (n=5), cancer of the central nervous system (n=3) & neuroendocrine (n=1).

Data collation – comorbidities:

Quan-Charlson indices (QCIs) were calculated using the weightings of Quan et. al. (18) but excluding cancer as a comorbidity. This was used to define 5-year comorbidities occurring prior to the patients’ Scottish Incidence date, as recorded in the cancer registry (https://www.ndc.scot.nhs.uk/National-Datasets/data.asp?ID=5&SubID=8). Total prescribed medicines within 1 year prior to consent were also extracted.

Analysis of COVID-19 PCR data and age correction

COVID-19 positive cases were defined as cases with a supporting positive PCR test. Publicly available population COVID-19 data was accessed from Public Health Scotland (PHS) data sources at (www.opendata.nhs.scot/dataset/covid-19-in-scotland) and monthly incident rates and cumulative total calculated for the combined local authorities in which the two hospital sites reside (the City of Edinburgh and West Lothian). Population COVID-19 infection rates were adjusted to per 1000 population values based on census data accessed from the National Records of Scotland (NRS - <https://www.ndc.scot.nhs.uk/National-Datasets/data.asp?ID=3&SubID=13>), or for cancer patients the total study size. As the ages of the patients in our cohort are all >25 years old, population COVID-19 data was age corrected to remove individuals under the age of 25. To do so, national data of daily COVID-19 infections, which is split by age groups 0-14, 15-19 and 20-24, were combined and compared. 10 year previous cancer treatments across the palliative group were calculated by grouping individual drug administrations into regimes. Only treatments >6 months prior to recruitment were considered.

Univariate and multivariate analysis

Univariate and multivariate analysis was carried out using the Survival package 3.2-13 in order to compute the Cox proportional hazards regression models. A multivariate model investigating the risk of catching COVID-19 during the study was defined as the length of time free from infection w.r.t recruitment (day) with patients without a COVID-19 positive PCR censored and the following variable binary groupings applied: Age > 60, gender = female, high socioeconomic score = SMID quintiles 4 & 5, High medication comorbidity > 5 prescribed medications in 1 year prior to recruitment, comorbidity QCI > 0 in 5 years prior to recruitment, vaccinated = 2 or more doses.

Stratification and analysis of COVID-19 antibody data

Patients were initially classified into 1 of 3 categories: i) no antibody (Ab) data available for patients (n=177; 23.1%), ii) Ab data only available prior to 14 days after 1st vaccination date (n= 242, 31.6%), iii) Ab data available >14d after 1st vaccination date (n=347, 45.3%).   Patients in group iii were then further categorised to determine the antibody response to vaccination into

1. Ab response - if the first antibody sample collected after vaccination 1 was reactive,
2. Delayed Ab response - if a single (or more) antibody sample collected after vaccination 1 was non-reactive but a later Ab sample was positive in the absence of PCR confirmed COVID-19.
3. Non-reactive after partial vaccination - if all antibody samples collected after vaccination 1 were non-reactive but samples were not collected 14 days after 2nd vaccination.
4. Non-reactive after full vaccination - if all antibody samples collected after vaccination 1 were non-reactive and samples were collected >14 days after 2nd vaccination.

To calculate antibody responses in total and split by treatment type, we considered cases in groups a, b and d, and calculated percentages against these values.To calculate COVID-19 prevalence between fully and partial/non vaccinated states, we considered all cases with at least 1 available antibody test (unvaccinated group only) and/or a positive PCR test.

**Supplemental figures**

| 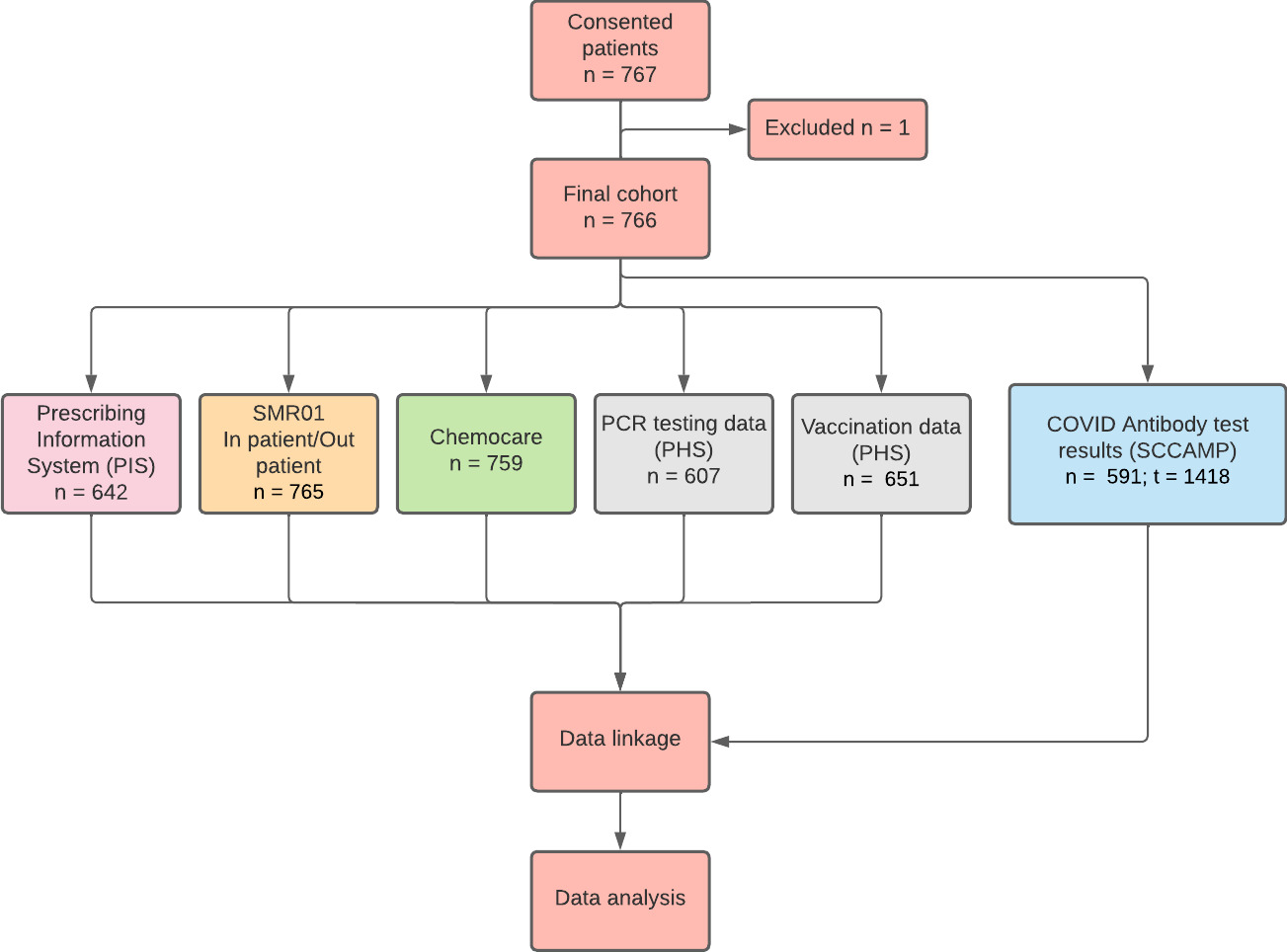 |
| --- |
| **Figure S1. Summary of data linkage in SCCAMP.** One patient was found to be diagnosed with concurrent haematological malignancy and excluded as per the exclusion criteria of the study. n = number of patients in each dataset. t = number of longitudinally collected antibody samples with processed data at time of data freeze. |

| **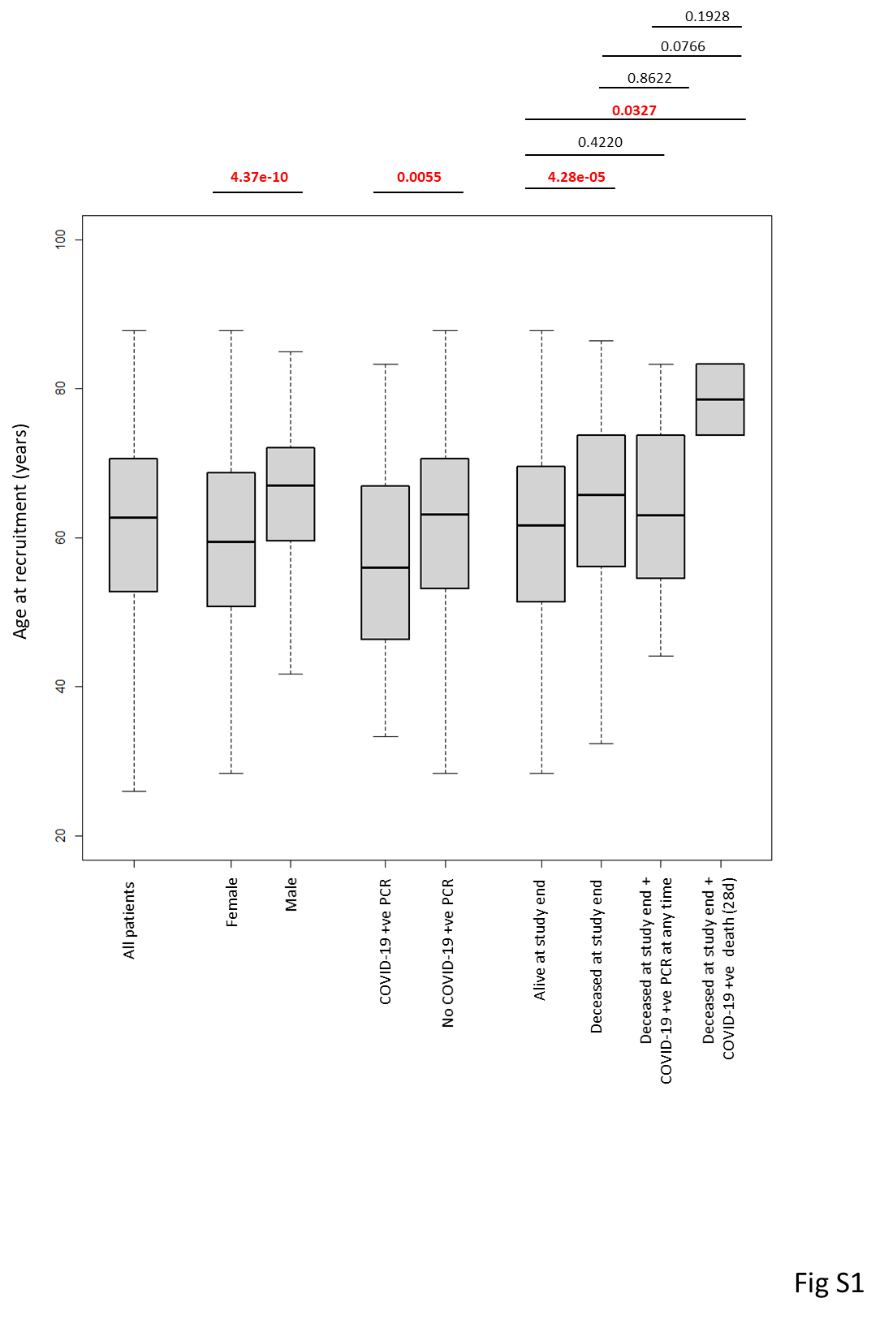** |
| --- |
| **Figure S2.** Boxplot of age of SCCAMP patients at recruitment split by a number of factors including gender, COVID-19 PCR status and survival outcome. P-values as calculated by pairwise Willcox tests are shown above with values in red reaching significance. |

| **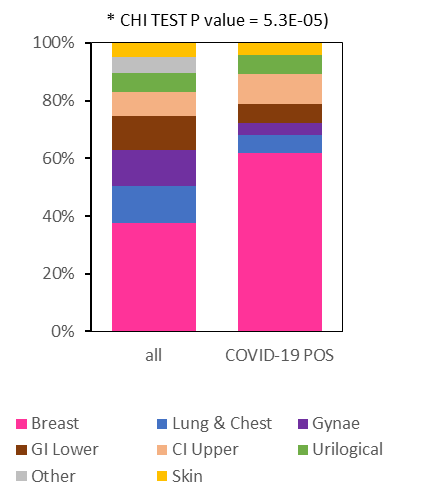** |
| --- |
| **Figure S3.** Stacked proportional bar plot of the cancer types present in the entire cohort (left) and those reported in patients with COVID-19 positive PCR. Chi squared test p-value comparing the relative proportion of cancer types between the two plots is shown above |
| **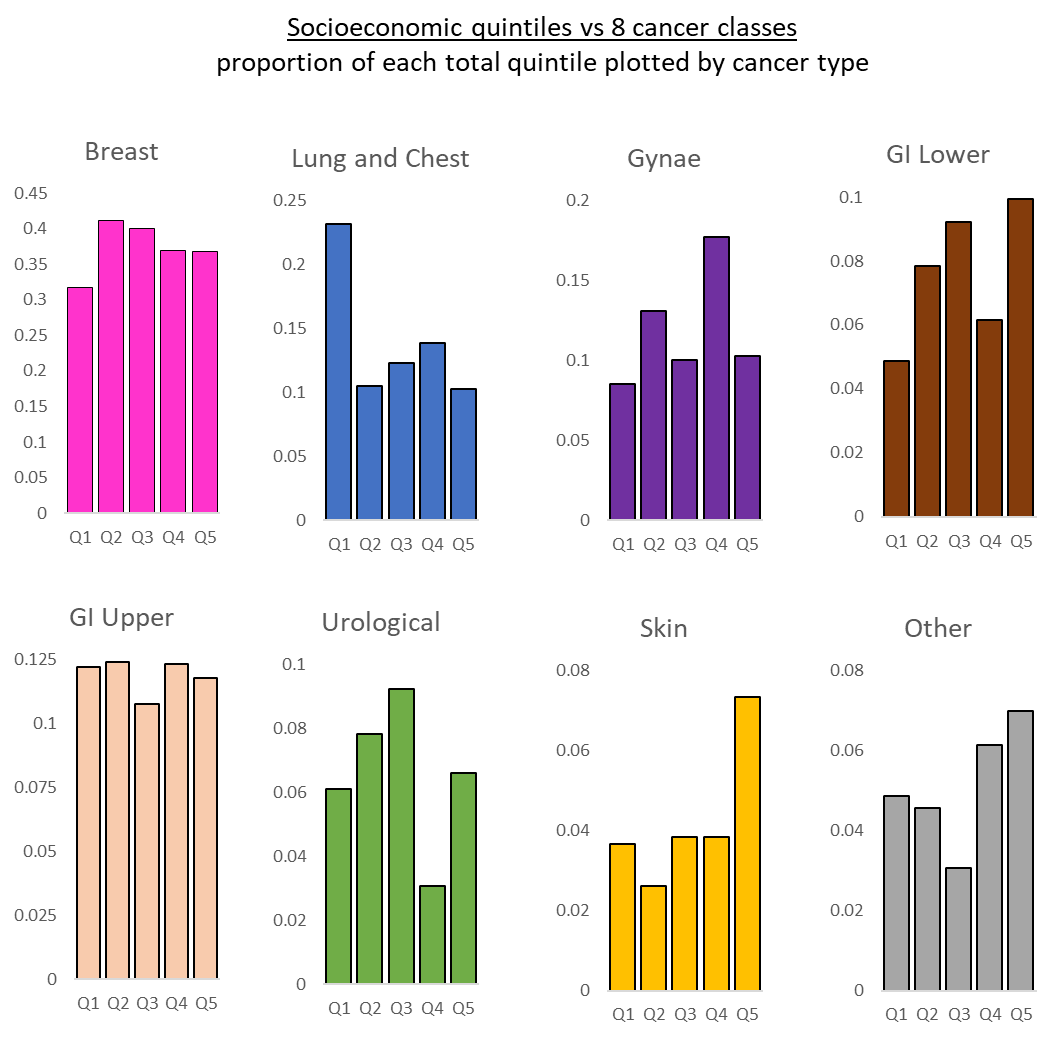** |
| **Figure S4.** Plots of proportions total SMID socioeconomic quintiles found in each of the 8 cancer classifications. |

| **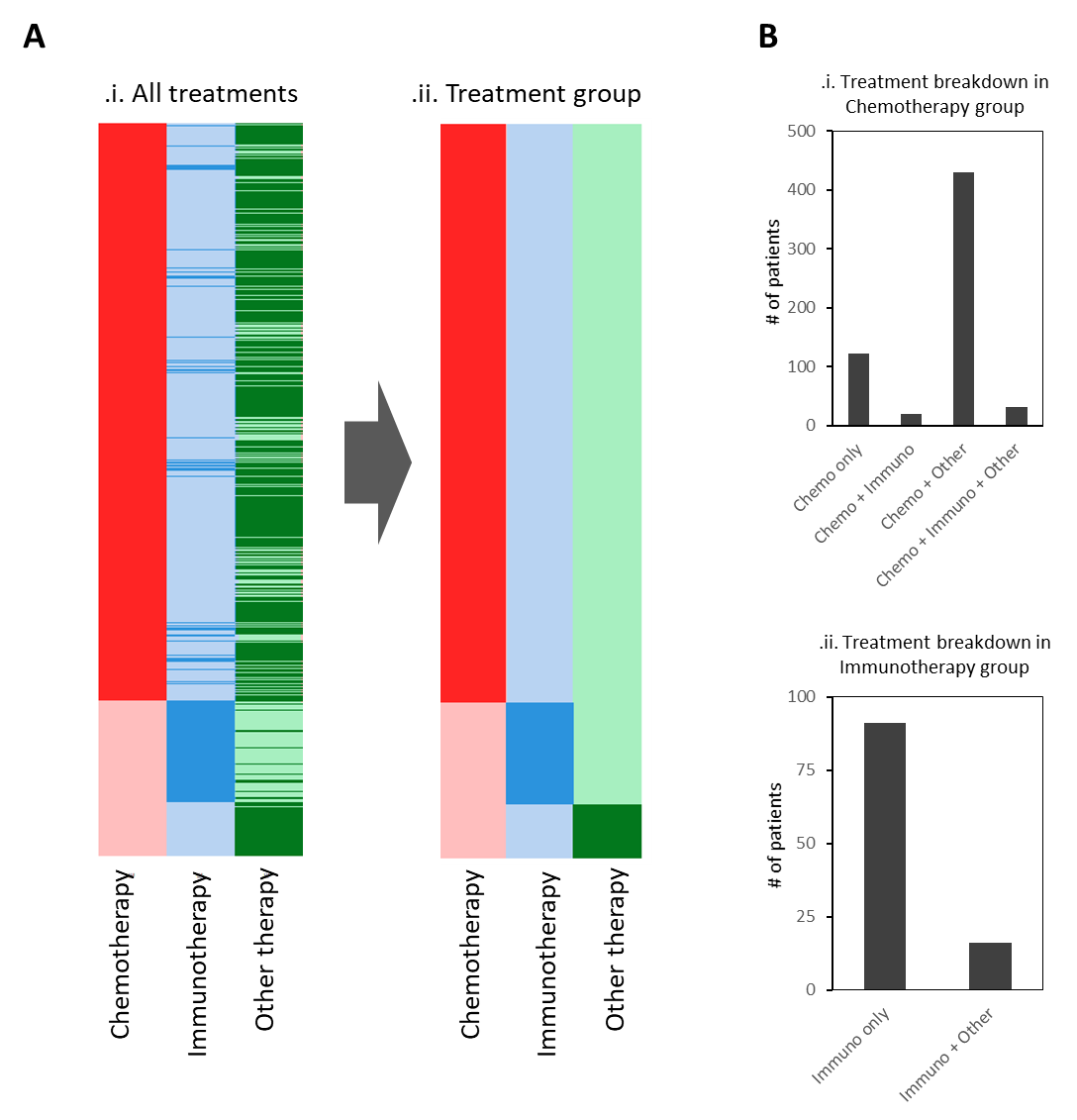** |
| --- |
| **Figure S5. A.** Heatmap plot of all treatment types across the 766 patients (i) as well as classified into the hierarchical treatment group (ii). **B.** Plots of treatment breakdown numbers for patients in the chemotherapy group (i) and immunotherapy group (ii). |

| **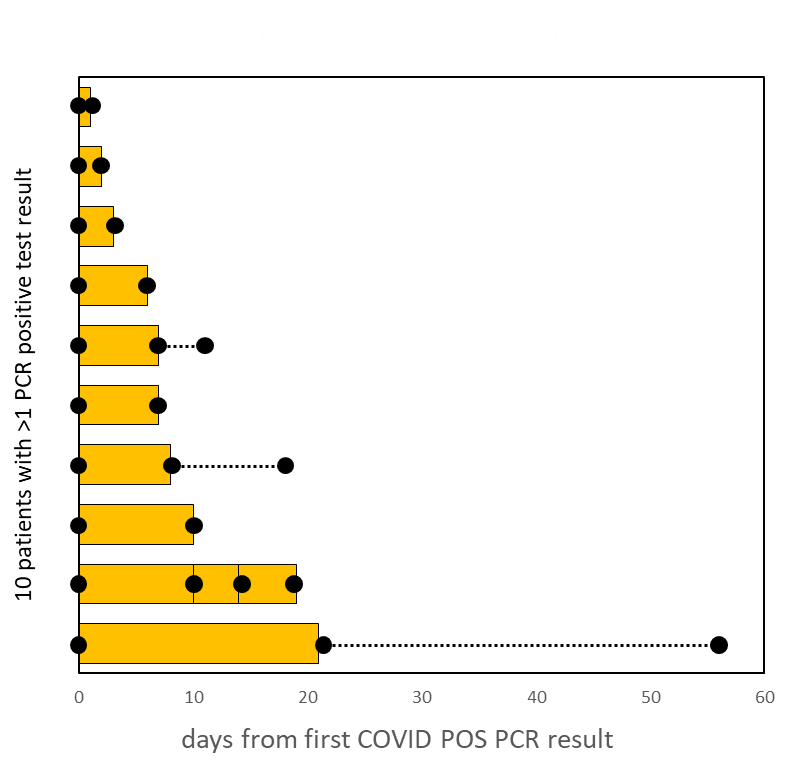** |
| --- |
| **Figure S6.** Plot of the number of days COVID-19 positive PCR result observed in patients with >1 positive PCR recorded. Black dots denote timing of PCR test. Dashed lines denote time between last positive test and first negative test. |

| **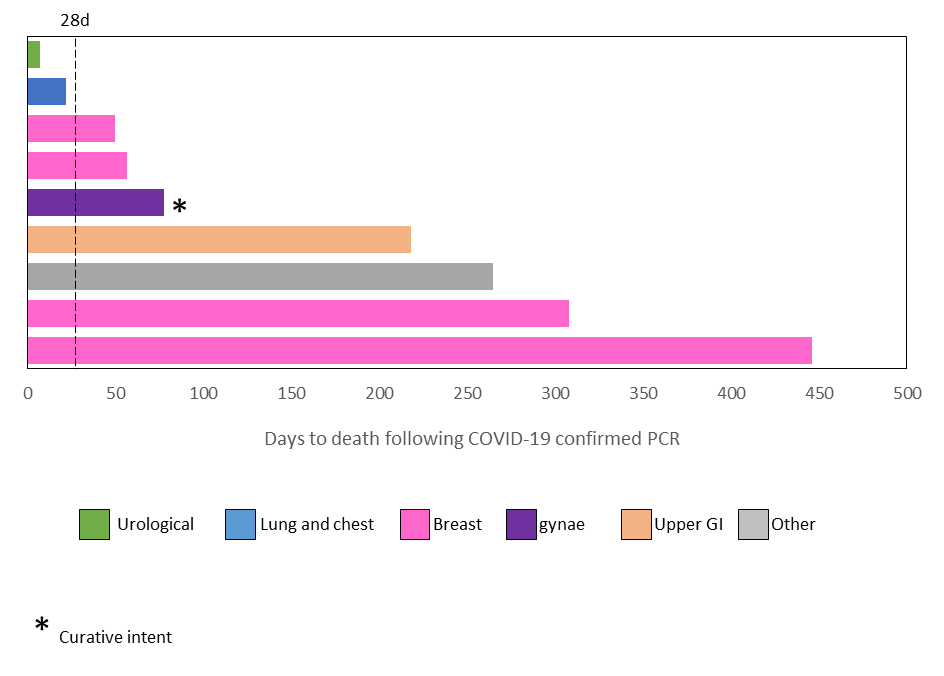** |
| --- |
| **Figure S7.** Plot of days to death in patients who were COVID-19 positive and then died during the study. Bars are colour coded by cancer type. Dashed bar denotes the classical classifier of a “COVID-19 death” set to 28 days after infection. Asterisk denotes curative intent. |

| **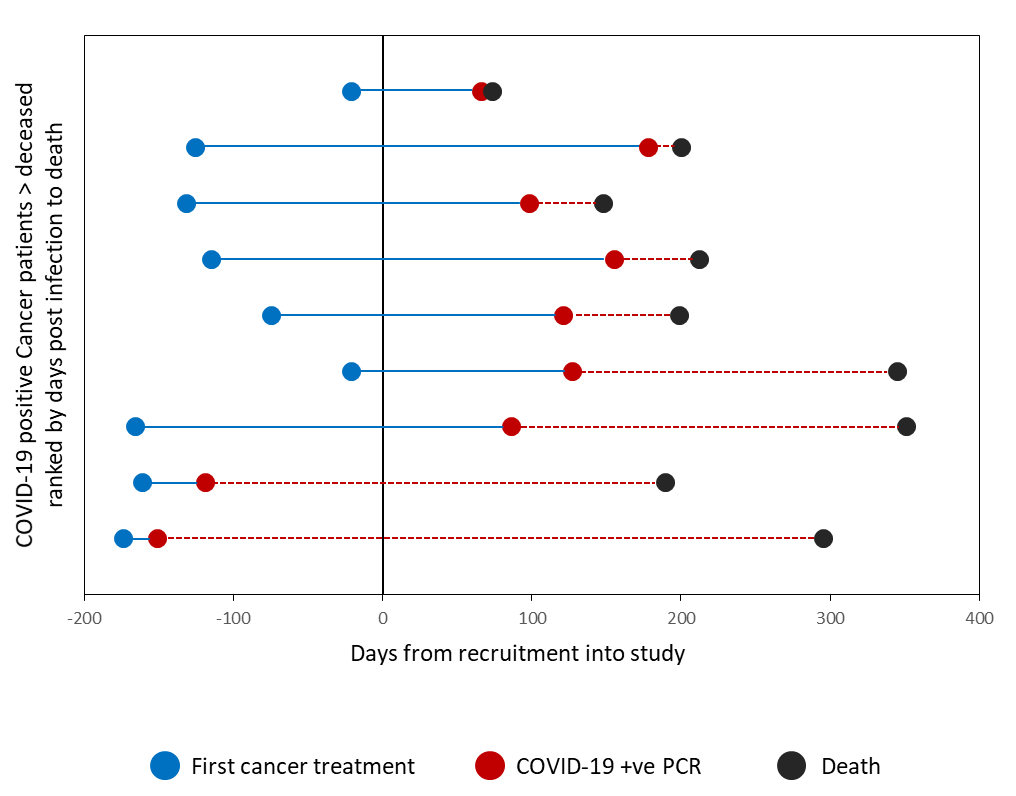** |
| --- |
| **Figure S8.** Plot of time between first cancer treatment (blue dots), COVID-19 infection (red dots) and death (black dots) across the 9 COVID-19 positive cancer patients in the study who went on to die before the study end. Plots are ranked by time between COVID-19 infection and death. Blue bars denote time between first treatment and infection, dashed red lines denote time between infection and death. Plots display time with respect to date of recruitment into the study. |

| 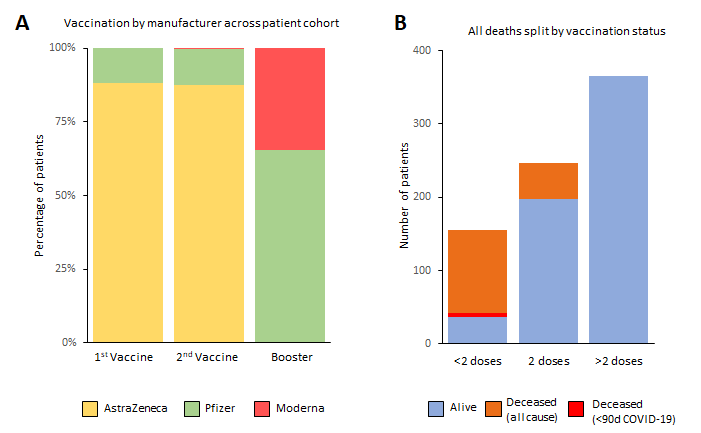 |
| --- |
| **Figure S9. A.** Stacked bar plot showing the percentages of the total cohort split by the manufacturer of the vaccine received at 1st dose, 2nd dose or booster. **B**. Plot of total vaccine doses per patient across the study with mortality data overlaid as a stacked plot. Alive = blue, deceased = orange, deceased within 90 days of confirmed Covid-19 infection = red. |

| 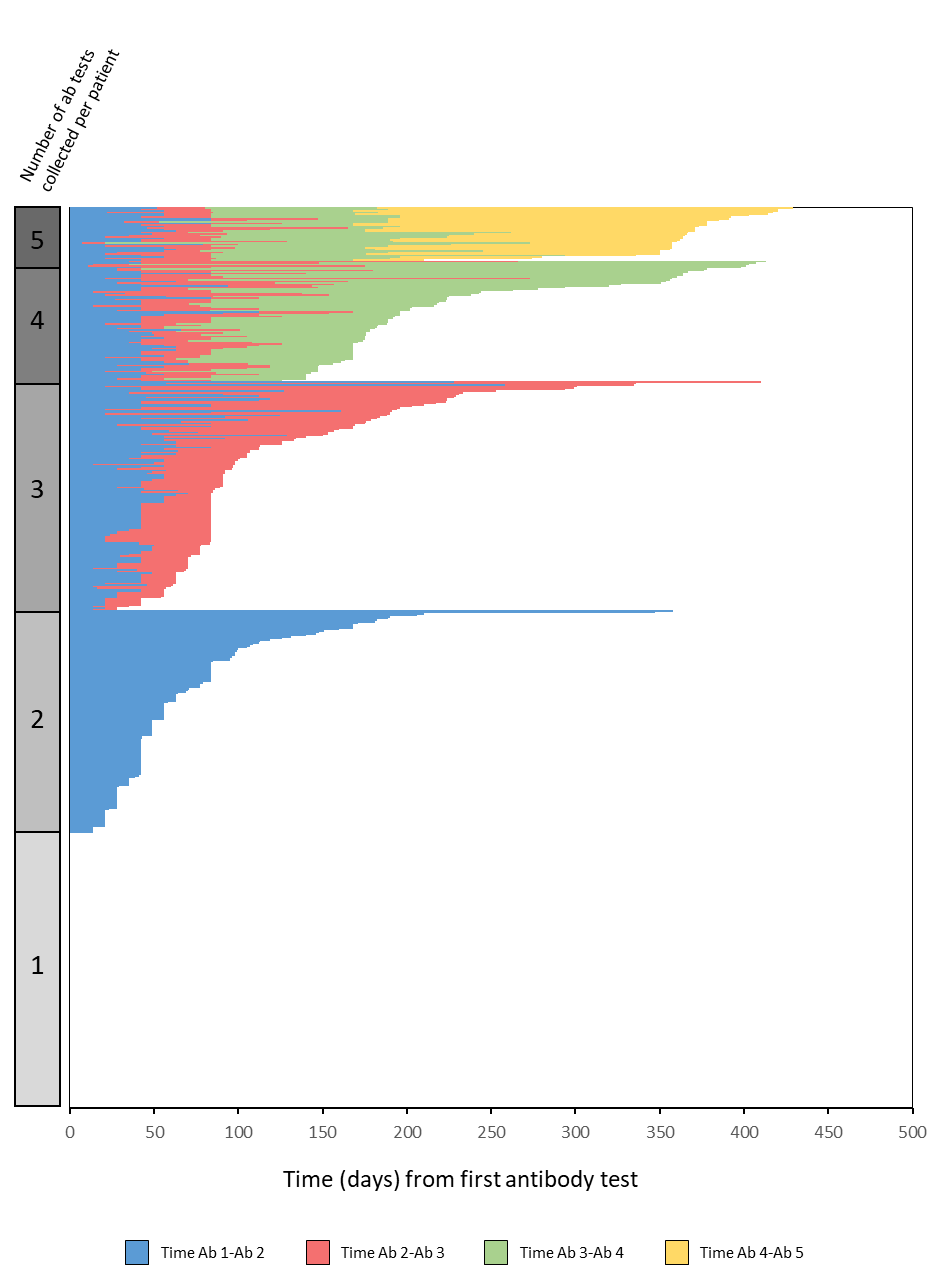 |
| --- |
| **Figure S10.** Plot of time differences between antibody test collections (days) for patients with available antibody data. Length of bars indicate time from previous collection. Note, no lines are visible for patients with only 1 antibody data point. Plot is ranked by length of time between antibody tests then ranked by number of collections. |

| **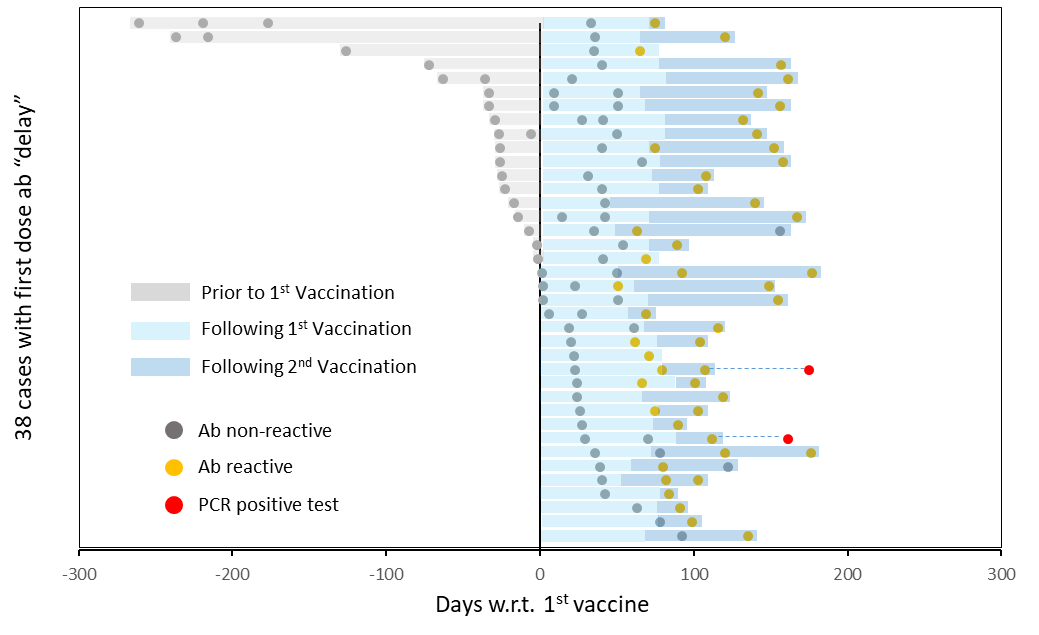** |
| --- |
| **Figure S11.** Plot of 38 cases displaying an initial non-reactive antibody result following vaccination 1, followed by a later conversion to reactive antibody state. All data is plotted with respect to (“w.r.t”) the date of vaccine 1. Dots denote antibody test collection dates. Grey dots = antibody non-reactive result, yellow dots = antibody reactive result, red dot = date of PCR positive test. Bars denote time w.r.t to vaccine dates. Grey bar = time prior to 1^st^ vaccination, light blue = time between 1^st^ vaccination and 2^nd^ vaccination (or last ab test), dark blue = time following 2^nd^ vaccination to last ab test. Dashed lines connecting to red dots denote time to PCR positive result. Cases are ranked by relative time to 1^st^ vaccine. |

| **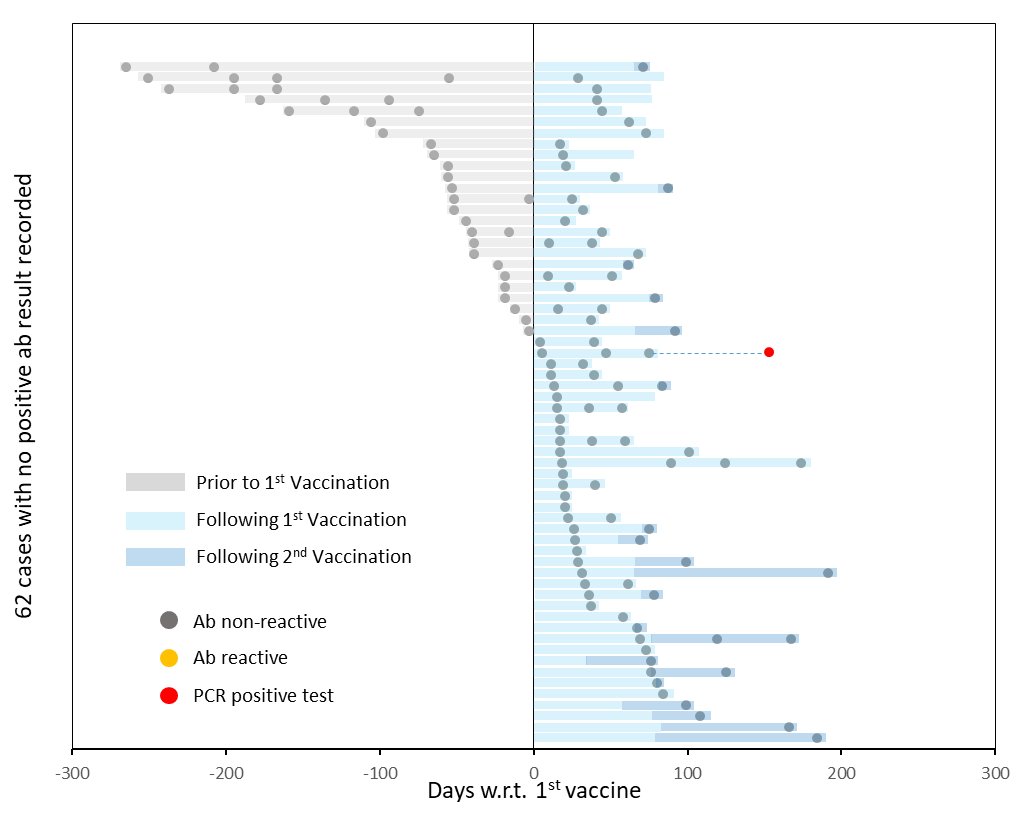** |
| --- |
| **Figure S12.** Plot of 62 cases displaying not reporting a reactive antibody result following vaccination 1. All data is plotted with respect to (“w.r.t”) the date of vaccine 1. Dots denote antibody test collection dates. Grey dots = antibody non-reactive result, yellow dots = antibody reactive result, red dot = date of PCR positive test. Bars denote time w.r.t to vaccine dates. Grey bar = time prior to 1^st^ vaccination, light blue = time between 1^st^ vaccination and 2^nd^ vaccination (or last ab test), dark blue = time following 2^nd^ vaccination to last ab test. Dashed lines connecting to red dots denote time to PCR positive result. Cases are ranked by relative time to 1^st^ vaccine. |

| **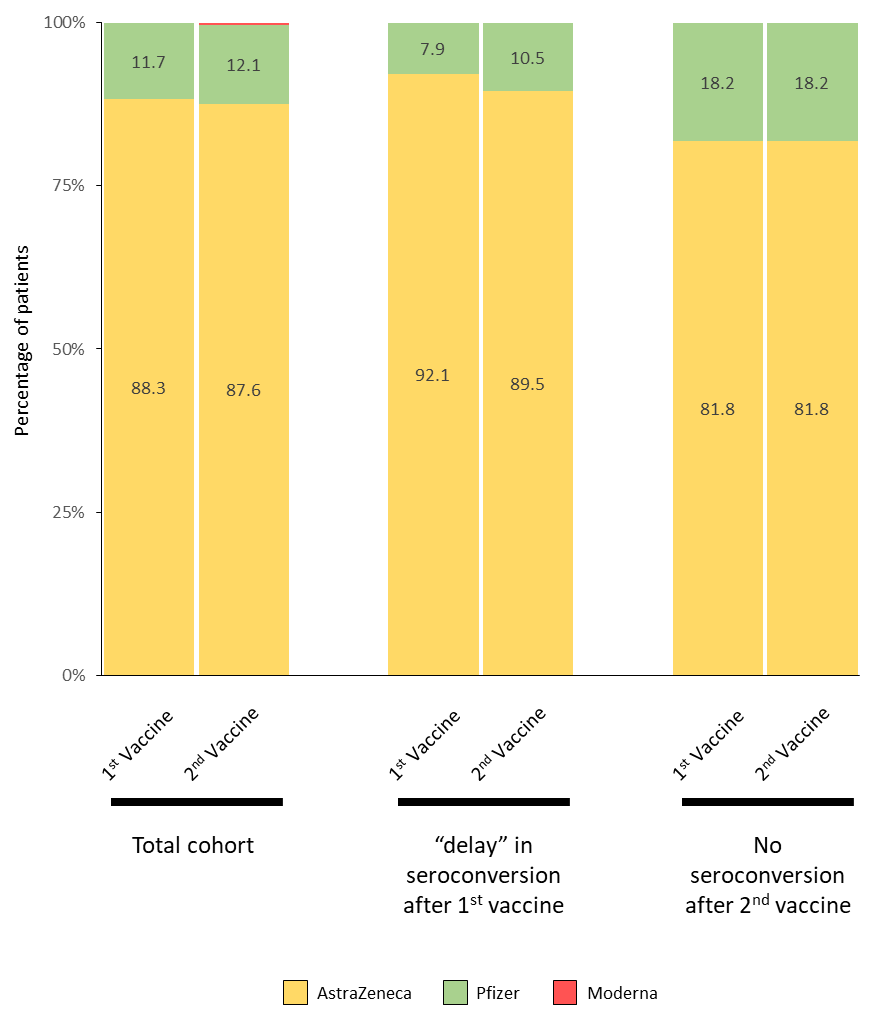** |
| --- |
| **Figure S13.** Stacked plots of percentage vaccine types split by manufacturer for the total cohort, patients displaying a “delayed” seroconversion following their first vaccination and patients displaying no seroconversion >14 days after their second vaccination. |
| **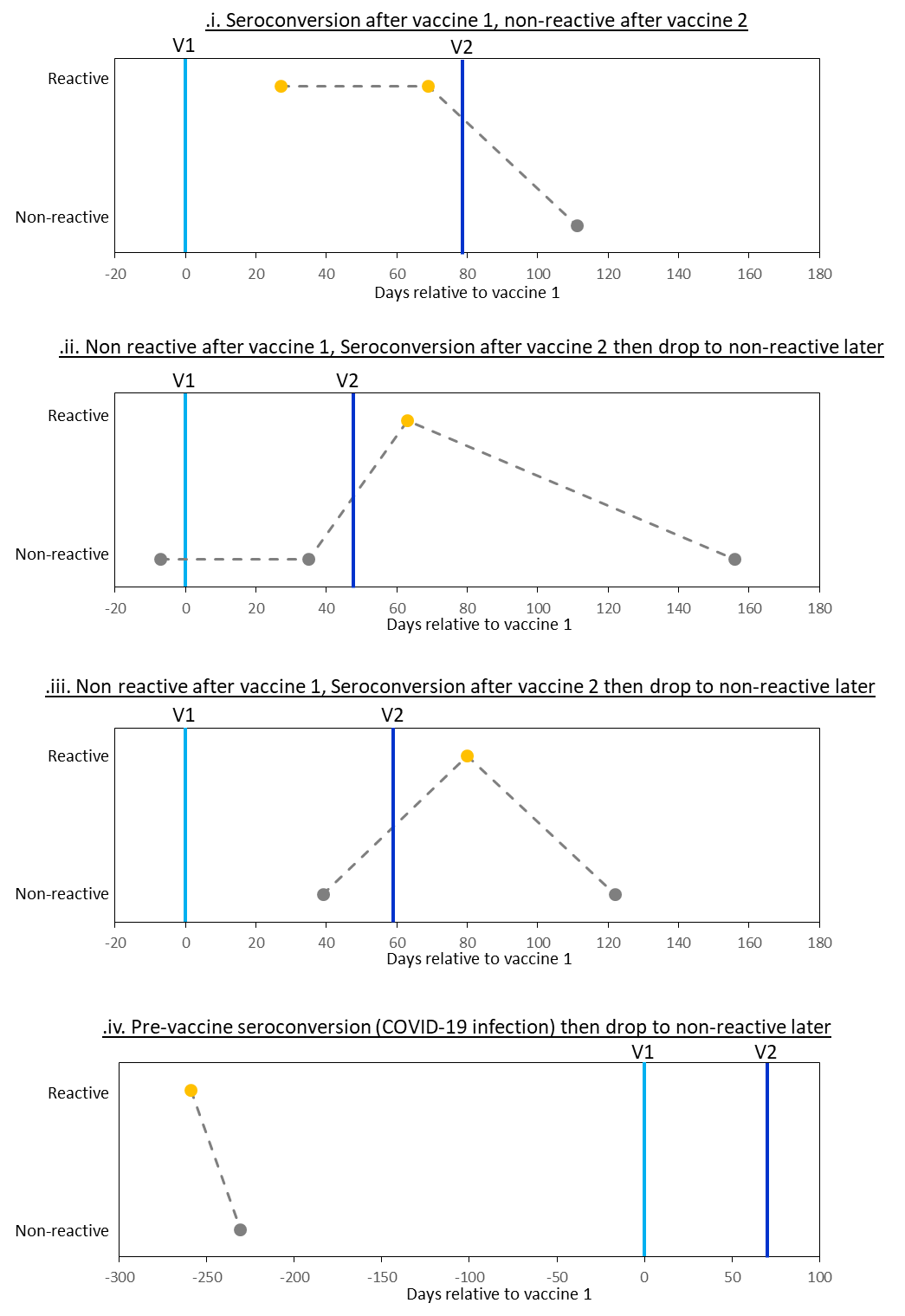** |
| **Figure S14.** Plots of 4 cases displaying sero-reversion at any time point in the study. Cases i,ii and iii represent post vaccination sero-reversion. Plots denote binary results (“reactive” and “non-reactive”) plotted relative to the date of their first vaccination (light blue line; day = 0). Vaccination time point 2 is denoted by a dark blue line. Grey dots denote non-reactive results, yellow dots denote reactive results. |
|  |
|  |

**Supplemental Tables**

| **CHEMO: Name of drug** | **IMMUNO: Name of drug** | **OTHER: Name of drug** |
| --- | --- | --- |
| CABAZITAXEL | 3WCarb/Etop/Atez | ABEMACICLIB |
| CAPECITABINE | ATEZOLIZ R1111 | ABIRATERONE |
| CARBOPLATIN | ATEZOLIZUMAB 948 | AXITINIB |
| CISPLATIN | AVELUMAB R891 | AZD1775 |
| CYCLOPHOSPHAMIDE | CEMIPLIMAB R922 | BEVACIZUMAB |
| DOCETAXEL | DURVALUMAB | CABOZANTINIB |
| DOXORUBICIN | IPI+NIVO R1217 | CETUXIMAB |
| DOXORUBICIN LIPOSOMAL (CAELYX) | NIVOLUMAB | CRIZOTINIB |
| EPIRUBICIN | PEMBROLIZUMAB | DABRAFENIB |
| ERIBULIN | T FAK-PD1 R831 | DEFACTINIB(VS-6063) |
| FLUOROURACIL | T LEAP-001 ARM 1 | DENOSUMAB |
| GEMCITABINE | T PRISM R900 | DS-8201A |
| HYDROXYCARBAMIDE |  | ENZALUTAMIDE |
| IRINOTECAN |  | ENZALUTAMIDE |
| METHOTREXATE |  | FULVESTRANT |
| NAB-PACLITAXEL (ABRAXANE) |  | IMATINIB |
| OXALIPLATIN |  | LENVATINIB |
| PACLITAXEL |  | LEVOTHYROXINE |
| PEMETREXED |  | NIRAPARIB |
| PROCARBAZINE |  | NUC-7738 |
| RALTITREXED |  | OLAPARIB |
| TOPOTECAN |  | OSIMERTINIB |
| VINCRISTINE |  | PALBOCICLIB |
| VINORELBINE |  | PANITUMUMAB |
|  |  | PAZOPANIB |
|  |  | RITUXIMAB |
|  |  | ROLAPITANT |
|  |  | RUCAPARIB |
|  |  | TIVOZANIB |
|  |  | TRAMETINIB |
|  |  | TRASTUZUMAB |
|  |  | TRASTUZUMAB EMTANSINE |
|  |  | TRASTUZUMAB(HERCEPTIN) |
|  |  | ZOLEDRONIC ACID |

Supplemental table S1. Table of drugs administered split by classification.

|  |  | **Curative intent** | | **Palliative intent** | |
| --- | --- | --- | --- | --- | --- |
|  | | **N** | **%** | **N** | **%** |
| **Cases** | Total | 325 | 42.4% total 766 cohort | 441 | 57.6% total 766 cohort |
| **Follow up period** | Median days | 388 | range 5-521 | 418 | range 3-521 |
| **Age at recruitment** | Median years | 57.4 | range 28.4-85.3 | 66.4 | range 26-87.8 |
| **Gender** | Female | 281 | 86.5 | 229 | 51.9 |
|  | Male | 44 | 13.5 | 212 | 51.6 |
|  |  |  |  |  |  |
| **Socioeconomic status (SIMD Quintiles)** | 1 | 34 | 10.5 | 48 | 10.9 |
|  | 2 | 71 | 21.8 | 82 | 18.6 |
|  | 3 | 52 | 16.0 | 78 | 17.7 |
|  | 4 | 57 | 17.5 | 72 | 16.3 |
|  | 5 | 111 | 34.2 | 161 | 36.5 |
| **Comorbidity: Quan-Charlson score ≤ 5yr** | 0 | 305 | 93.8 | 385 | 87.3 |
|  | 1 | 13 | 4.0 | 38 | 8.6 |
|  | 2 | 5 | 1.5 | 15 | 3.4 |
|  | ≥3 | 2 | 0.6 | 3 | 0.7 |
| **Comorbidity: Prescribed meds ≤ 1 yr** | Median prescribed meds | 4 | range 0-28 | 5 | range 0-28 |
|  |  |  |  |  |  |
| **Vaccination status at end of study or at death** | ≤1 | 29 | 8.9 | 126 | 28.6 |
|  | ≥2 | 296 | 91.1 | 315 | 71.4 |
| **PCR confirmed COVID-19** | Yes [<6M prior to treatment] | 2 | 0.6 | 3 | 0.7 |
|  | Yes (during treatment) | 21 | 6.5 | 22 | 5.0 |
|  | No | 302 | 92.9 | 416 | 94.3 |
|  |  |  |  |  |  |
| **chemotherapy** | Yes | 287 | 88.3 | 316 | 71.6 |
| **immunotherapy; no chemotherapy** | Yes | 27 | 8.3 | 81 | 18.4 |
| **Other treatment** | Yes | 12 | 3.7 | 44 | 10.0 |
|  |  |  |  |  |  |
| **Death details** | Died within 28d of PCR confirmed COVID-19 : Unvaccinated prior to COVID | 0 | 0 [0% COVID-19 positive curative patients, n=23] | 2 | 0.5 [8% COVID-19 positive palliative patients, n=25] |
|  | Died within 28d of PCR confirmed COVID-19 : Vaccinated prior to COVID | 0 | 0 [0% COVID-19 positive curative patients, n=23] | 0 | 0 [0% COVID-19 positive palliative patients, n=25] |
|  | Died within 90d of PCR confirmed COVID-19 : Unvaccinated prior to COVID | 0 | 0 [0% COVID-19 positive curative patients, n=23] | 2 | 0.5 [8% COVID-19 positive palliative patients, n=25] |
|  | Died within 90d of PCR confirmed COVID-19 : Vaccinated prior to COVID | 0 | 0 [0% COVID-19 positive curative patients, n=23] | 0 | 0 [0% COVID-19 positive palliative patients, n=25] |
|  | Died < 2 years after PCR confirmed COVID-19 : Unvaccinated prior to COVID | 1 | 0.3 [4% COVID-19 positive curative patients, n=23] | 8 | 0.9 [32% COVID-19 positive palliative patients, n=25] |
|  | Died < 2 years after PCR confirmed COVID-19 : Vaccinated prior to COVID | 0 | 0 [0% COVID-19 positive curative patients, n=23] | 0 | 0 [0% COVID-19 positive palliative patients, n=25] |
|  | Died, no PCR confirmed COVID-19 | 20 | 6.2 | 138 | 31.3 |
|  | Alive at end of study | 304 | 93.5 [91% COVID-19 positive curative patients, n=23] | 295 | 66.9 [68% COVID-19 positive palliative patients, n=25] |

Supplemental table S2. SCCAMP cohort metrics, split by curative intent.

|  |  | **Palliative intent** | |
| --- | --- | --- | --- |
|  | | **N** | **%** |
| **Cases** | Total | 441 | 57.6% total 766 cohort |
|  | Age at recruitment | 66.4 | range 26-87.8 |
|  | Female | 229 | 51.9 |
|  | Male | 212 | 51.6 |
|  |  |  |  |
| **10 year previous cancer treatments** | Median Previous 10 year treatments | 2 | range 0-8 |
|  | previous treatment(s): 0 | 172 | 39.0% |
|  | previous treatment(s): 1 | 96 | 21.8% |
|  | previous treatment(s): 2 | 72 | 16.3% |
|  | previous treatment(s): 3 | 38 | 8.6% |
|  | previous treatment(s): >3 | 62 | 14.1% |

Supplemental table S3. Metrics for 10 year previous cancer treatments across SCCAMP palliative patients. Treatments are taken 6 months prior to recruitment.

|  |  | Prev meds = 0 | Prev meds 1-5 | Prev meds >5 |
| --- | --- | --- | --- | --- |
| Recruits | Number of patients | 124 | 313 | 329 |
|  |  |  |  |  |
| COVID PCR | Positive PCR | 6 [4.8%] | 20 [6.4%] | 22 [6.7%] |
|  | No Positive PCR | 118 [95.2%] | 293 [93.6%] | 307 [93.3%] |
|  |  |  |  |  |
| vaccination | ≤1 | 40 [32.3%] | 49 [15.7%] | 66 [20.1%] |
|  | ≥2 | 84 [67.7%] | 264 [84.3%] | 263 [79.9%] |
|  |  |  |  |  |
| Treatment intent | Curative | 65 [52.4%] | 134 [42.8%] | 126 [38.3%] |
|  | Palliative | 59 [47.6%] | 179 [57.2%] | 203 [61.7%] |
|  |  |  |  |  |
| Deaths | Within 28d COVID | 1 [0.8%] | 0 | 1 [0.3%] |
|  | Within 90d COVID | 2 [1.6%] | 0 | 3 [0.9%] |
|  | Died any time after COVID | 2 [1.6%] | 2 [0.6%] | 5 [1.5%] |
|  | Died, no PCR confirmed COVID-19 | 21 [16.9%] | 61 [19.5%] | 76 [23.1%] |

Supplemental table S4. SCCAMP cohort metrics, split by numbers of previous medicines, 1 year prior to recruitment.


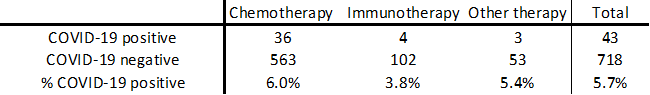


Supplemental table S5. COVID-19 PCR rates per treatment type
